# Supplementary material for: Altered microRNA Transcriptome in Cultured Human Liver Cells upon Infection with Ebola Virus
Source: Int J Mol Sci. 2021 Apr 6;22(7):3792. doi: 10.3390/ijms22073792 (PMC8038836; doi:10.3390/ijms22073792)
Supplement: Supplementary file 1 [file ijms-22-03792-s001.zip › Supplementary_File/C_ GO_Analysis_Results/16-30nt_go_Makona-96h-Huh7_vs_Control-96h-Huh7_up.mature_mirna_targets/BP_result(Human).html]

| GO.ID | Term | Ontology | Count | Pop.Hits | List.Total | Pop.Total | Fold.Enrichment | Pvalue | FDR | Enrichment.Score | Gene.Ratio | GENES |
| --- | --- | --- | --- | --- | --- | --- | --- | --- | --- | --- | --- | --- |
| GO:0012501 | programmed cell death | Biological process | 34 | 2088 | 143 | 17653 | 2.01016129464405 | 4.67358990249478e-05 | 0.188843617570015 | 4.33034939868265 | 0.237762237762238 | BAK1//NOD1//DHCR24//KIF1B//FAS//LTA//PDE1B//HIF3A//MUL1//SLTM//ZC3H12A//TRAF4//PAX7//PRKAA2//RARB//SMO//PRDX2//THBS1//GCLM//MAP3K8//PPP1R13B//ALX3//ALX4//NCOA1//RASSF2//GFRAL//RIPK3//KRT6C//ST14//TCHH//CUL2//VNN1//CD274// |
| GO:0008219 | cell death | Biological process | 35 | 2216 | 143 | 17653 | 1.94975827421676 | 6.58460732572676e-05 | 0.188843617570015 | 4.18147011916157 | 0.244755244755245 | BAK1//NOD1//DHCR24//KIF1B//FAS//LTA//PDE1B//HIF3A//MUL1//SLTM//ZC3H12A//TRAF4//PAX7//PRKAA2//RARB//SMO//PRDX2//THBS1//GCLM//MAP3K8//PPP1R13B//ALX3//ALX4//NCOA1//RASSF2//GFRAL//RIPK3//KRT6C//ST14//TCHH//CUL2//VNN1//CD274//PMP22// |
| GO:0035116 | embryonic hindlimb morphogenesis | Biological process | 4 | 29 | 143 | 17653 | 17.0272486134555 | 8.37691634940182e-05 | 0.188843617570015 | 4.07691582133384 | 0.027972027972028 | ALX3//AFF3//RARB//ALX4// |
| GO:0006915 | apoptotic process | Biological process | 31 | 1958 | 143 | 17653 | 1.95448116745359 | 0.000183560552749869 | 0.299634365158109 | 3.73622064319869 | 0.216783216783217 | BAK1//NOD1//FAS//MUL1//DHCR24//PAX7//PRKAA2//RARB//SMO//PRDX2//THBS1//GCLM//ZC3H12A//MAP3K8//PPP1R13B//ALX3//ALX4//TRAF4//LTA//NCOA1//RASSF2//GFRAL//RIPK3//CUL2//VNN1//CD274//KIF1B//PDE1B//HIF3A//SLTM// |
| GO:0035137 | hindlimb morphogenesis | Biological process | 4 | 37 | 143 | 17653 | 13.3456813456813 | 0.000221524741355988 | 0.299634365158109 | 3.65457776183676 | 0.027972027972028 | ALX3//AFF3//RARB//ALX4// |
| GO:0042981 | regulation of apoptotic process | Biological process | 26 | 1566 | 143 | 17653 | 2.04957622199002 | 0.000324450439277163 | 0.320334398441912 | 3.48885163350856 | 0.181818181818182 | NOD1//FAS//MUL1//DHCR24//PAX7//PRKAA2//RARB//SMO//PRDX2//THBS1//BAK1//ZC3H12A//LTA//NCOA1//RASSF2//GCLM//GFRAL//MITF//RIPK3//PPP1R13B//VNN1//CD274//MAP3K8//ALX3//ALX4//TRAF4// |
| GO:0043067 | regulation of programmed cell death | Biological process | 26 | 1579 | 143 | 17653 | 2.03270194023836 | 0.000368690644367388 | 0.320334398441912 | 3.43333788260318 | 0.181818181818182 | NOD1//FAS//MUL1//DHCR24//PAX7//PRKAA2//RARB//SMO//PRDX2//THBS1//BAK1//ZC3H12A//MAP3K8//PPP1R13B//ALX3//ALX4//TRAF4//LTA//NCOA1//RASSF2//GCLM//GFRAL//MITF//RIPK3//VNN1//CD274// |
| GO:0045017 | glycerolipid biosynthetic process | Biological process | 9 | 272 | 143 | 17653 | 4.08466166186754 | 0.000378925800315732 | 0.320334398441912 | 3.42144582345743 | 0.0629370629370629 | ETNK2//DGKA//AGPAT4//PIP5K1C//MTM1//PIP5K1A//SYNJ1//MOGAT3//PCK1// |
| GO:0002544 | chronic inflammatory response | Biological process | 3 | 19 | 143 | 17653 | 19.4917188075083 | 0.000458601366013152 | 0.344613448705216 | 3.33856465599271 | 0.020979020979021 | LTA//THBS1//VNN1// |
| GO:0031098 | stress-activated protein kinase signaling cascade | Biological process | 9 | 296 | 143 | 17653 | 3.75347287847288 | 0.000696539343944964 | 0.38997652404448 | 3.15705434747317 | 0.0629370629370629 | NOD1//MUL1//FAS//TRAF4//RASSF2//MAP3K8//ZC3H12A//KAT7//GFRAL// |
| GO:0009595 | detection of biotic stimulus | Biological process | 3 | 22 | 143 | 17653 | 16.833757151939 | 0.000715988125379906 | 0.38997652404448 | 3.14509418038034 | 0.020979020979021 | NOD1//PGLYRP4//SMO// |
| GO:0031638 | zymogen activation | Biological process | 4 | 51 | 143 | 17653 | 9.68216097627862 | 0.000767824114340647 | 0.38997652404448 | 3.11473825251997 | 0.027972027972028 | THBS1//DHCR24//ST14//BAK1// |
| GO:0042493 | response to drug | Biological process | 18 | 966 | 143 | 17653 | 2.30026495243887 | 0.000818271046526197 | 0.38997652404448 | 3.0871028155065 | 0.125874125874126 | PDE1B//FKBP1A//GJB2//THBS1//TRPC3//PRKAA2//TFRC//BAK1//NPPC//NOD1//PCK1//KAT7//GCLM//ZC3H12A//KIF1B//NCOA1//LTA//TIMP2// |
| GO:0032649 | regulation of interferon-gamma production | Biological process | 5 | 91 | 143 | 17653 | 6.78283255206332 | 0.000864948670806921 | 0.38997652404448 | 3.06300966438113 | 0.034965034965035 | CD274//HLA-DPB1//LTA//ZC3H12A//RIPK3// |
| GO:0097327 | response to antineoplastic agent | Biological process | 5 | 91 | 143 | 17653 | 6.78283255206332 | 0.000864948670806921 | 0.38997652404448 | 3.06300966438113 | 0.034965034965035 | GJB2//PCK1//KAT7//ZC3H12A//KIF1B// |
| GO:0010941 | regulation of cell death | Biological process | 26 | 1696 | 143 | 17653 | 1.89247427101201 | 0.00107657379141178 | 0.455054284457367 | 2.96795619704846 | 0.181818181818182 | NOD1//FAS//MUL1//DHCR24//PAX7//PRKAA2//RARB//SMO//PRDX2//THBS1//BAK1//ZC3H12A//MAP3K8//PPP1R13B//ALX3//ALX4//TRAF4//LTA//NCOA1//RASSF2//GCLM//GFRAL//MITF//RIPK3//VNN1//CD274// |
| GO:0031639 | plasminogen activation | Biological process | 3 | 26 | 143 | 17653 | 14.243948359333 | 0.00118051027639663 | 0.469634764662965 | 2.92793022801522 | 0.020979020979021 | THBS1//DHCR24//ST14// |
| GO:0035107 | appendage morphogenesis | Biological process | 6 | 149 | 143 | 17653 | 4.97104238043835 | 0.0013525617410403 | 0.481440792350292 | 2.86884290131854 | 0.041958041958042 | BAK1//ALX3//HOXA9//ALX4//AFF3//RARB// |
| GO:0035108 | limb morphogenesis | Biological process | 6 | 149 | 143 | 17653 | 4.97104238043835 | 0.0013525617410403 | 0.481440792350292 | 2.86884290131854 | 0.041958041958042 | ALX3//HOXA9//ALX4//AFF3//RARB//BAK1// |
| GO:0032609 | interferon-gamma production | Biological process | 5 | 102 | 143 | 17653 | 6.05135061017414 | 0.00144311195722031 | 0.487988308334048 | 2.84069997485462 | 0.034965034965035 | RIPK3//CD274//HLA-DPB1//LTA//ZC3H12A// |
| GO:0043065 | positive regulation of apoptotic process | Biological process | 13 | 627 | 143 | 17653 | 2.55951863128897 | 0.00177078616738151 | 0.563596569619165 | 2.75183387920129 | 0.0909090909090909 | NOD1//FAS//MUL1//BAK1//ZC3H12A//PPP1R13B//THBS1//CD274//RIPK3//LTA//RARB//NCOA1//RASSF2// |
| GO:1900182 | positive regulation of protein localization to nucleus | Biological process | 4 | 65 | 143 | 17653 | 7.59677245831092 | 0.00190612238711486 | 0.563596569619165 | 2.7198492178938 | 0.027972027972028 | SMO//ZC3H12A//NMD3//KAT7// |
| GO:0043068 | positive regulation of programmed cell death | Biological process | 13 | 633 | 143 | 17653 | 2.53525779118196 | 0.00192539929481077 | 0.563596569619165 | 2.71547919158383 | 0.0909090909090909 | NOD1//FAS//MUL1//BAK1//LTA//RARB//NCOA1//RASSF2//RIPK3//ZC3H12A//PPP1R13B//THBS1//CD274// |
| GO:0000041 | transition metal ion transport | Biological process | 5 | 110 | 143 | 17653 | 5.61125238397966 | 0.0020141329699766 | 0.563596569619165 | 2.69591186137881 | 0.034965034965035 | TRPC3//TRPC5//TFRC//ATP6V0E1//SLC30A2// |
| GO:0070887 | cellular response to chemical stimulus | Biological process | 40 | 3175 | 143 | 17653 | 1.55524475524476 | 0.00208338226238047 | 0.563596569619165 | 2.68123103768008 | 0.27972027972028 | NCOA1//BAK1//FKBP1A//ATP6V0E1//RARB//THBS1//STX1A//PRDX2//TMEM100//NR6A1//RNF4//NCOA4//PIP5K1C//PCK1//FAS//LTA//GJB2//GPX3//ZC3H12A//PRKAA2//TFRC//GCLM//DLL4//PDE1B//TMEM199//PIP5K1A//HLA-DPB1//MUL1//HIF3A//CUL2//NOD1//MAP3K8//CD274//SLC41A1//CUX2//PTPN7//SMO//VNN1//KIF1B//LAPTM5// |
| GO:0034329 | cell junction assembly | Biological process | 7 | 223 | 143 | 17653 | 3.87503527862272 | 0.00228951332118941 | 0.574095017630693 | 2.64025682525171 | 0.048951048951049 | CDH24//TNS1//GJB2//PIP5K1C//WDR1//PIP5K1A//THBS1// |
| GO:0008654 | phospholipid biosynthetic process | Biological process | 8 | 287 | 143 | 17653 | 3.44104675811993 | 0.00237419751021405 | 0.574095017630693 | 2.62448315478831 | 0.0559440559440559 | ETNK2//DGKA//AGPAT4//PIP5K1C//MTM1//PIP5K1A//SYNJ1//IDI2// |
| GO:0035115 | embryonic forelimb morphogenesis | Biological process | 3 | 33 | 143 | 17653 | 11.2225047679593 | 0.00237685354039027 | 0.574095017630693 | 2.62399757828864 | 0.020979020979021 | ALX3//HOXA9//ALX4// |
| GO:0034097 | response to cytokine | Biological process | 19 | 1164 | 143 | 17653 | 2.01503736813015 | 0.00271155440892834 | 0.631079927077447 | 2.56678167676779 | 0.132867132867133 | FKBP1A//STX1A//FAS//LTA//AFF3//PDE1B//HLA-DPB1//MUL1//NOD1//MAP3K8//PCK1//PTPN7//ZC3H12A//THBS1//GCLM//TFRC//LAPTM5//CD274//TIMP2// |
| GO:0006828 | manganese ion transport | Biological process | 2 | 10 | 143 | 17653 | 24.6895104895105 | 0.00281004536698692 | 0.631079927077447 | 2.55128666853868 | 0.013986013986014 | TRPC3//TRPC5// |
| GO:0042098 | T cell proliferation | Biological process | 6 | 174 | 143 | 17653 | 4.25681215336388 | 0.00295448064770936 | 0.631079927077447 | 2.52951885036889 | 0.041958041958042 | CD274//CD209//HLA-DPB1//TFRC//RIPK3//FKBP1A// |
| GO:0048736 | appendage development | Biological process | 6 | 176 | 143 | 17653 | 4.20843928798474 | 0.00312610497736815 | 0.631079927077447 | 2.5049964420794 | 0.041958041958042 | BAK1//ALX3//HOXA9//ALX4//AFF3//RARB// |
| GO:0060173 | limb development | Biological process | 6 | 176 | 143 | 17653 | 4.20843928798474 | 0.00312610497736815 | 0.631079927077447 | 2.5049964420794 | 0.041958041958042 | BAK1//ALX3//HOXA9//ALX4//AFF3//RARB// |
| GO:0019219 | regulation of nucleobase-containing compound metabolic process | Biological process | 50 | 4328 | 143 | 17653 | 1.42615009759187 | 0.00320217564385734 | 0.631079927077447 | 2.49455485009757 | 0.34965034965035 | CUX2//NR6A1//MITF//DLL4//H2AFY2//PURB//RARB//SMO//NCOA1//KAT7//ZNF597//ZNF320//KDM2A//AFF3//YBX2//PRKAA2//TAF11//ZNF329//LZTR1//ZNF251//WDHD1//FOXP4//ALX3//QRICH1//HIF3A//SLTM//CREB5//BAK1//USP51//EBF3//RNF4//NCOA4//FUBP3//ZNF488//TMEM100//NPPC//THBS1//NOD1//ZC3H12A//CDK12//TIMP2//TFRC//HOXA9//PAX7//ALX4//RIPK3//NOVA2//PCK1//CUL2//NMD3// |
| GO:0044650 | adhesion of symbiont to host cell | Biological process | 2 | 11 | 143 | 17653 | 22.4450095359186 | 0.0034163016983757 | 0.631079927077447 | 2.466443783137 | 0.013986013986014 | CLEC4M//CD209// |
| GO:0019048 | modulation by virus of host morphology or physiology | Biological process | 3 | 38 | 143 | 17653 | 9.74585940375414 | 0.00356828087170777 | 0.631079927077447 | 2.44754096813226 | 0.020979020979021 | ZC3H12A//CLEC4M//CD209// |
| GO:0010942 | positive regulation of cell death | Biological process | 13 | 681 | 143 | 17653 | 2.35656120678147 | 0.00361070006939764 | 0.631079927077447 | 2.44240858568436 | 0.0909090909090909 | NOD1//FAS//MUL1//BAK1//LTA//RARB//NCOA1//RASSF2//RIPK3//ZC3H12A//PPP1R13B//THBS1//CD274// |
| GO:0046474 | glycerophospholipid biosynthetic process | Biological process | 7 | 243 | 143 | 17653 | 3.55610233388011 | 0.00368572609228718 | 0.631079927077447 | 2.43347694286309 | 0.048951048951049 | ETNK2//DGKA//AGPAT4//PIP5K1C//MTM1//PIP5K1A//SYNJ1// |
| GO:0048522 | positive regulation of cellular process | Biological process | 57 | 5142 | 143 | 17653 | 1.36843844603471 | 0.00371838942826335 | 0.631079927077447 | 2.42964512836476 | 0.398601398601399 | MAP3K8//NOD1//THBS1//NCOA1//FAS//CDK2AP1//STX1A//SMO//MUL1//BAK1//TRAF4//RARB//TRPC5//PRKAA2//ZC3H12A//TRPC3//RIPK3//LARP4B//EBF3//MITF//RNF4//NCOA4//FUBP3//CREB5//CD274//MCAM//NPPC//WDR1//TFRC//PAX7//HLA-DPB1//FKBP1A//RASSF2//VNN1//F2RL2//CD209//LTA//TIMP2//TMEM100//H2AFY2//DLL4//KAT7//NR6A1//HOXA9//CDK12//ALX4//HIF3A//ZNF488//CUX2//PGLYRP4//ACPP//SLC30A2//PCK1//PPP1R13B//NMD3//NBPF3//SYNJ1// |
| GO:0014070 | response to organic cyclic compound | Biological process | 16 | 937 | 143 | 17653 | 2.10796247509161 | 0.00383891609769003 | 0.631079927077447 | 2.41579137960138 | 0.111888111888112 | NR6A1//RNF4//NCOA4//NCOA1//FKBP1A//GJB2//THBS1//TRPC3//RARB//PCK1//MUL1//SMO//GCLM//ZC3H12A//KIF1B//BAK1// |
| GO:0030326 | embryonic limb morphogenesis | Biological process | 5 | 129 | 143 | 17653 | 4.7847888545563 | 0.00400918784546853 | 0.631079927077447 | 2.39694359494947 | 0.034965034965035 | ALX3//HOXA9//ALX4//AFF3//RARB// |
| GO:0035113 | embryonic appendage morphogenesis | Biological process | 5 | 129 | 143 | 17653 | 4.7847888545563 | 0.00400918784546853 | 0.631079927077447 | 2.39694359494947 | 0.034965034965035 | ALX3//HOXA9//ALX4//AFF3//RARB// |
| GO:2001233 | regulation of apoptotic signaling pathway | Biological process | 9 | 383 | 143 | 17653 | 2.90085632383283 | 0.00403783749154749 | 0.631079927077447 | 2.39385116383329 | 0.0629370629370629 | BAK1//FAS//PPP1R13B//MUL1//THBS1//VNN1//GCLM//GFRAL//RIPK3// |
| GO:0035136 | forelimb morphogenesis | Biological process | 3 | 41 | 143 | 17653 | 9.03274774006482 | 0.00443004340442583 | 0.631079927077447 | 2.35359201865046 | 0.020979020979021 | ALX3//HOXA9//ALX4// |
| GO:0010761 | fibroblast migration | Biological process | 3 | 42 | 143 | 17653 | 8.81768231768232 | 0.00474282818724759 | 0.631079927077447 | 2.32396260772886 | 0.020979020979021 | THBS1//TNS1//PIP5K1A// |
| GO:0051646 | mitochondrion localization | Biological process | 3 | 42 | 143 | 17653 | 8.81768231768232 | 0.00474282818724759 | 0.631079927077447 | 2.32396260772886 | 0.020979020979021 | KIF1B//MTM1//MUL1// |
| GO:0016045 | detection of bacterium | Biological process | 2 | 13 | 143 | 17653 | 18.9919311457773 | 0.00479377858456693 | 0.631079927077447 | 2.31932202906779 | 0.013986013986014 | NOD1//PGLYRP4// |
| GO:0019835 | cytolysis | Biological process | 3 | 43 | 143 | 17653 | 8.61261993820133 | 0.0050686413771694 | 0.631079927077447 | 2.29510843543395 | 0.020979020979021 | PGLYRP4//LYZL6//BAK1// |
| GO:0071383 | cellular response to steroid hormone stimulus | Biological process | 7 | 260 | 143 | 17653 | 3.32358795051103 | 0.0053160059624589 | 0.631079927077447 | 2.27441454062217 | 0.048951048951049 | NR6A1//RNF4//NCOA4//NCOA1//RARB//GJB2//PCK1// |
| GO:0036295 | cellular response to increased oxygen levels | Biological process | 2 | 14 | 143 | 17653 | 17.6353646353646 | 0.00556316441824907 | 0.631079927077447 | 2.25467810438003 | 0.013986013986014 | FAS//TMEM199// |
| GO:0044406 | adhesion of symbiont to host | Biological process | 2 | 14 | 143 | 17653 | 17.6353646353646 | 0.00556316441824907 | 0.631079927077447 | 2.25467810438003 | 0.013986013986014 | CLEC4M//CD209// |
| GO:0098543 | detection of other organism | Biological process | 2 | 14 | 143 | 17653 | 17.6353646353646 | 0.00556316441824907 | 0.631079927077447 | 2.25467810438003 | 0.013986013986014 | NOD1//PGLYRP4// |
| GO:0051403 | stress-activated MAPK cascade | Biological process | 7 | 267 | 143 | 17653 | 3.23645268589089 | 0.00612537886450076 | 0.631079927077447 | 2.21286704432286 | 0.048951048951049 | NOD1//MUL1//FAS//TRAF4//RASSF2//ZC3H12A//MAP3K8// |
| GO:0044003 | modification by symbiont of host morphology or physiology | Biological process | 3 | 46 | 143 | 17653 | 8.05092733353603 | 0.00612570399477553 | 0.631079927077447 | 2.21284399292636 | 0.020979020979021 | CLEC4M//CD209//ZC3H12A// |
| GO:0003417 | growth plate cartilage development | Biological process | 2 | 15 | 143 | 17653 | 16.4596736596737 | 0.00638511048378035 | 0.631079927077447 | 2.19483158358756 | 0.013986013986014 | NPPC//RARB// |
| GO:0016082 | synaptic vesicle priming | Biological process | 2 | 15 | 143 | 17653 | 16.4596736596737 | 0.00638511048378035 | 0.631079927077447 | 2.19483158358756 | 0.013986013986014 | STX1A//SYNJ1// |
| GO:1990000 | amyloid fibril formation | Biological process | 2 | 15 | 143 | 17653 | 16.4596736596737 | 0.00638511048378035 | 0.631079927077447 | 2.19483158358756 | 0.013986013986014 | RIPK3//FKBP1A// |
| GO:0006919 | activation of cysteine-type endopeptidase activity involved in apoptotic process | Biological process | 4 | 91 | 143 | 17653 | 5.42626604165066 | 0.00640231132630466 | 0.631079927077447 | 2.19366321116521 | 0.027972027972028 | BAK1//NOD1//FAS//MUL1// |
| GO:0009791 | post-embryonic development | Biological process | 4 | 91 | 143 | 17653 | 5.42626604165066 | 0.00640231132630466 | 0.631079927077447 | 2.19366321116521 | 0.027972027972028 | BAK1//NPPC//ETNK2//ALX4// |
| GO:0048518 | positive regulation of biological process | Biological process | 62 | 5841 | 143 | 17653 | 1.31034895595758 | 0.00642468389880767 | 0.631079927077447 | 2.19214823521545 | 0.433566433566434 | MAP3K8//NOD1//THBS1//NCOA1//FAS//CDK2AP1//STX1A//SMO//PGLYRP4//CD209//ZC3H12A//LTA//MUL1//BAK1//TRAF4//RARB//TRPC5//PRKAA2//TRPC3//KIF1B//CUX2//MITF//DLL4//FUBP3//RIPK3//EDIL3//LARP4B//CLTCL1//EBF3//RNF4//NCOA4//CREB5//HLA-DPB1//CD274//MCAM//NPPC//WDR1//TFRC//PAX7//FKBP1A//RASSF2//VNN1//F2RL2//TIMP2//TAF11//TMEM100//H2AFY2//KAT7//NR6A1//HOXA9//CDK12//ALX4//HIF3A//MTM1//ZNF488//ACPP//SLC30A2//PCK1//PPP1R13B//NMD3//NBPF3//SYNJ1// |
| GO:0034330 | cell junction organization | Biological process | 7 | 270 | 143 | 17653 | 3.2004921004921 | 0.00649910972809087 | 0.631079927077447 | 2.1871461305353 | 0.048951048951049 | CDH24//TNS1//GJB2//PIP5K1C//WDR1//PIP5K1A//THBS1// |
| GO:1901028 | regulation of mitochondrial outer membrane permeabilization involved in apoptotic signaling pathway | Biological process | 3 | 47 | 143 | 17653 | 7.87963100729058 | 0.0065050529308291 | 0.631079927077447 | 2.18674916528633 | 0.020979020979021 | PPP1R13B//BAK1//MUL1// |
| GO:0043588 | skin development | Biological process | 9 | 413 | 143 | 17653 | 2.69014036810647 | 0.00654465375844159 | 0.631079927077447 | 2.18411332463283 | 0.0629370629370629 | ALX4//ST14//PIP5K1A//SMO//KRT6C//KRTAP4-4//H2AFY2//TCHH//DHCR24// |
| GO:0035690 | cellular response to drug | Biological process | 8 | 340 | 143 | 17653 | 2.90464829288359 | 0.00655469798448036 | 0.631079927077447 | 2.18344731414863 | 0.0559440559440559 | NOD1//GJB2//PCK1//GCLM//ZC3H12A//NCOA1//PRKAA2//TFRC// |
| GO:0071396 | cellular response to lipid | Biological process | 11 | 569 | 143 | 17653 | 2.38650804380154 | 0.00664431539999506 | 0.631079927077447 | 2.17754976014203 | 0.0769230769230769 | NR6A1//RNF4//NCOA4//NCOA1//RARB//CD274//ZC3H12A//PCK1//PRKAA2//SMO//GJB2// |
| GO:0050830 | defense response to Gram-positive bacterium | Biological process | 4 | 92 | 143 | 17653 | 5.36728488902402 | 0.00665201748305512 | 0.631079927077447 | 2.17704661802372 | 0.027972027972028 | NOD1//LTA//PGLYRP4//LYZL6// |
| GO:0045935 | positive regulation of nucleobase-containing compound metabolic process | Biological process | 24 | 1742 | 143 | 17653 | 1.70076995335319 | 0.00670591656954228 | 0.631079927077447 | 2.17354185407046 | 0.167832167832168 | NCOA1//BAK1//EBF3//MITF//RNF4//NCOA4//FUBP3//CREB5//NPPC//TIMP2//PRKAA2//TFRC//KAT7//NR6A1//HOXA9//PAX7//CDK12//RARB//ALX4//HIF3A//SMO//ZC3H12A//PCK1//NMD3// |
| GO:0051173 | positive regulation of nitrogen compound metabolic process | Biological process | 36 | 2965 | 143 | 17653 | 1.49885729784549 | 0.00681854697200457 | 0.631079927077447 | 2.16630816336009 | 0.251748251748252 | MAP3K8//NOD1//THBS1//NCOA1//FAS//CDK2AP1//MUL1//BAK1//TRAF4//TRPC5//LARP4B//EBF3//MITF//RNF4//NCOA4//FUBP3//CREB5//NPPC//PAX7//FKBP1A//RASSF2//RIPK3//TIMP2//PRKAA2//TFRC//KAT7//NR6A1//HOXA9//CDK12//RARB//ALX4//HIF3A//SMO//ZC3H12A//PCK1//NMD3// |
| GO:0017001 | antibiotic catabolic process | Biological process | 3 | 48 | 143 | 17653 | 7.71547202797203 | 0.00689811123204492 | 0.631079927077447 | 2.16126980691356 | 0.020979020979021 | PCK1//GPX3//PRDX2// |
| GO:0042982 | amyloid precursor protein metabolic process | Biological process | 3 | 48 | 143 | 17653 | 7.71547202797203 | 0.00689811123204492 | 0.631079927077447 | 2.16126980691356 | 0.020979020979021 | NECAB3//DHCR24//FKBP1A// |
| GO:0070231 | T cell apoptotic process | Biological process | 3 | 48 | 143 | 17653 | 7.71547202797203 | 0.00689811123204492 | 0.631079927077447 | 2.16126980691356 | 0.020979020979021 | RIPK3//BAK1//CD274// |
| GO:0071407 | cellular response to organic cyclic compound | Biological process | 11 | 572 | 143 | 17653 | 2.37399139322216 | 0.0069008751396465 | 0.631079927077447 | 2.16109583039042 | 0.0769230769230769 | NR6A1//RNF4//NCOA4//NCOA1//RARB//PCK1//MUL1//SMO//GJB2//GCLM//ZC3H12A// |
| GO:0048535 | lymph node development | Biological process | 2 | 16 | 143 | 17653 | 15.4309440559441 | 0.00725872692324147 | 0.631079927077447 | 2.13913954165431 | 0.013986013986014 | RIPK3//LTA// |
| GO:1902001 | fatty acid transmembrane transport | Biological process | 2 | 16 | 143 | 17653 | 15.4309440559441 | 0.00725872692324147 | 0.631079927077447 | 2.13913954165431 | 0.013986013986014 | PRKAA2//THBS1// |
| GO:0042129 | regulation of T cell proliferation | Biological process | 5 | 149 | 143 | 17653 | 4.14253531703196 | 0.00732966321230387 | 0.631079927077447 | 2.13491598011989 | 0.034965034965035 | CD274//CD209//HLA-DPB1//TFRC//RIPK3// |
| GO:0019725 | cellular homeostasis | Biological process | 15 | 916 | 143 | 17653 | 2.02152105536385 | 0.00740788237118712 | 0.631079927077447 | 2.13030592237768 | 0.104895104895105 | PRDX2//TMEM199//TFRC//NCOA4//ATP6V0E1//TRPC5//BAK1//TRPC3//SLC41A1//MTM1//FKBP1A//F2RL2//SLC30A2//GCLM//PCK1// |
| GO:0001775 | cell activation | Biological process | 20 | 1373 | 143 | 17653 | 1.7982163502921 | 0.00745149143805847 | 0.631079927077447 | 2.12775679322584 | 0.13986013986014 | BAK1//DGKA//F2RL2//TRPC3//DLL4//TFRC//MAP3K8//CD274//HLA-DPB1//RIPK3//VNN1//GCLM//FKBP1A//CD209//HOXA9//ZC3H12A//THBS1//ACPP//TIMP2//SMO// |
| GO:0045595 | regulation of cell differentiation | Biological process | 24 | 1761 | 143 | 17653 | 1.68241979485591 | 0.00764217780367458 | 0.631079927077447 | 2.11678286219113 | 0.167832167832168 | SMO//PAX7//PMP22//RARB//VNN1//ZC3H12A//TMEM100//H2AFY2//PURB//HOXA9//THBS1//TIMP2//NCOA1//RASSF2//NPPC//MITF//TRPC5//WDR1//ZNF488//DLL4//CUX2//LTA//MUL1//CDK12// |
| GO:0042102 | positive regulation of T cell proliferation | Biological process | 4 | 96 | 143 | 17653 | 5.14364801864802 | 0.00771525123247254 | 0.631079927077447 | 2.11264992739733 | 0.027972027972028 | CD274//CD209//HLA-DPB1//TFRC// |
| GO:0032874 | positive regulation of stress-activated MAPK cascade | Biological process | 5 | 151 | 143 | 17653 | 4.08766729958783 | 0.00774257311523434 | 0.631079927077447 | 2.11111468479031 | 0.034965034965035 | MUL1//NOD1//TRAF4//RASSF2//ZC3H12A// |
| GO:0050870 | positive regulation of T cell activation | Biological process | 6 | 213 | 143 | 17653 | 3.47739584359303 | 0.00782929051643876 | 0.631079927077447 | 2.10627759155077 | 0.041958041958042 | MAP3K8//CD274//HLA-DPB1//VNN1//CD209//TFRC// |
| GO:0070304 | positive regulation of stress-activated protein kinase signaling cascade | Biological process | 5 | 152 | 143 | 17653 | 4.06077475156423 | 0.00795498437333071 | 0.631079927077447 | 2.09936066913907 | 0.034965034965035 | MUL1//NOD1//TRAF4//RASSF2//ZC3H12A// |
| GO:0035821 | modification of morphology or physiology of other organism | Biological process | 5 | 153 | 143 | 17653 | 4.03423374011609 | 0.00817141628938251 | 0.631079927077447 | 2.087702663987 | 0.034965034965035 | CLEC4M//CD209//PGLYRP4//TAF11//ZC3H12A// |
| GO:0002577 | regulation of antigen processing and presentation | Biological process | 2 | 17 | 143 | 17653 | 14.5232414644179 | 0.0081831346705794 | 0.631079927077447 | 2.08708030154195 | 0.013986013986014 | THBS1//NOD1// |
| GO:0017085 | response to insecticide | Biological process | 2 | 17 | 143 | 17653 | 14.5232414644179 | 0.0081831346705794 | 0.631079927077447 | 2.08708030154195 | 0.013986013986014 | ZC3H12A//KIF1B// |
| GO:0010604 | positive regulation of macromolecule metabolic process | Biological process | 37 | 3106 | 143 | 17653 | 1.47056002593672 | 0.00820219108000013 | 0.631079927077447 | 2.08607011751342 | 0.258741258741259 | MAP3K8//NOD1//THBS1//NCOA1//FAS//CDK2AP1//MUL1//BAK1//TRAF4//KIF1B//CUX2//MITF//DLL4//SMO//ZC3H12A//FUBP3//TRPC5//LARP4B//EBF3//RNF4//NCOA4//CREB5//PAX7//FKBP1A//RASSF2//RIPK3//TIMP2//TFRC//KAT7//NR6A1//HOXA9//CDK12//RARB//ALX4//HIF3A//PCK1//NMD3// |
| GO:0032872 | regulation of stress-activated MAPK cascade | Biological process | 6 | 216 | 143 | 17653 | 3.42909867909868 | 0.00835808215010813 | 0.631079927077447 | 2.07789336456792 | 0.041958041958042 | MUL1//NOD1//TRAF4//RASSF2//ZC3H12A//FAS// |
| GO:0043281 | regulation of cysteine-type endopeptidase activity involved in apoptotic process | Biological process | 6 | 216 | 143 | 17653 | 3.42909867909868 | 0.00835808215010813 | 0.631079927077447 | 2.07789336456792 | 0.041958041958042 | NOD1//FAS//MUL1//BAK1//DHCR24//THBS1// |
| GO:0050769 | positive regulation of neurogenesis | Biological process | 9 | 430 | 143 | 17653 | 2.5837859814604 | 0.00841900812334631 | 0.631079927077447 | 2.07473907143759 | 0.0629370629370629 | SMO//RARB//TIMP2//NCOA1//TRPC5//ZNF488//CUX2//LTA//MUL1// |
| GO:0010033 | response to organic substance | Biological process | 38 | 3218 | 143 | 17653 | 1.45773989838626 | 0.00842613220016261 | 0.631079927077447 | 2.0743717313769 | 0.265734265734266 | PDE1B//NCOA1//THBS1//GPX3//BAK1//FKBP1A//ATP6V0E1//DHCR24//TIMP2//RARB//STX1A//TMEM100//NR6A1//RNF4//NCOA4//GJB2//FAS//LTA//PCK1//TRPC3//CD274//AFF3//GCLM//DLL4//NPPC//HLA-DPB1//MUL1//NOD1//MAP3K8//ZC3H12A//CUX2//PTPN7//PRKAA2//SMO//KAT7//KIF1B//TFRC//LAPTM5// |
| GO:0070302 | regulation of stress-activated protein kinase signaling cascade | Biological process | 6 | 217 | 143 | 17653 | 3.41329638103832 | 0.00853991952210259 | 0.631079927077447 | 2.06854622196573 | 0.041958041958042 | MUL1//FAS//NOD1//TRAF4//RASSF2//ZC3H12A// |
| GO:0009893 | positive regulation of metabolic process | Biological process | 39 | 3329 | 143 | 17653 | 1.44621644501488 | 0.00858485188394575 | 0.631079927077447 | 2.0662671934023 | 0.272727272727273 | MAP3K8//NOD1//THBS1//NCOA1//FAS//CDK2AP1//MUL1//BAK1//TRAF4//PRKAA2//ZC3H12A//KIF1B//CUX2//MITF//DLL4//SMO//FUBP3//RIPK3//TRPC5//LARP4B//EBF3//RNF4//NCOA4//CREB5//NPPC//PAX7//FKBP1A//RASSF2//TIMP2//TFRC//KAT7//NR6A1//HOXA9//CDK12//RARB//ALX4//HIF3A//PCK1//NMD3// |
| GO:2001141 | regulation of RNA biosynthetic process | Biological process | 43 | 3774 | 143 | 17653 | 1.40653014182426 | 0.00904591751319441 | 0.645124354950502 | 2.04354737676342 | 0.300699300699301 | CUX2//NR6A1//MITF//DLL4//H2AFY2//PURB//RARB//SMO//NCOA1//KAT7//ZNF597//ZNF320//KDM2A//AFF3//YBX2//PRKAA2//TAF11//ZNF329//LZTR1//ZNF251//WDHD1//FOXP4//ALX3//QRICH1//HIF3A//SLTM//CREB5//EBF3//RNF4//NCOA4//FUBP3//ZNF488//TMEM100//NOD1//ZC3H12A//HOXA9//PAX7//CDK12//ALX4//RIPK3//PCK1//CUL2//NMD3// |
| GO:0046068 | cGMP metabolic process | Biological process | 3 | 53 | 143 | 17653 | 6.98759730835202 | 0.00907299590121108 | 0.645124354950502 | 2.04224928531823 | 0.020979020979021 | NPPC//THBS1//PDE1B// |
| GO:0006071 | glycerol metabolic process | Biological process | 2 | 18 | 143 | 17653 | 13.7163947163947 | 0.00915746533716519 | 0.645124354950502 | 2.03822471655164 | 0.013986013986014 | PCK1//MOGAT3// |
| GO:0032026 | response to magnesium ion | Biological process | 2 | 18 | 143 | 17653 | 13.7163947163947 | 0.00915746533716519 | 0.645124354950502 | 2.03822471655164 | 0.013986013986014 | SLC41A1//THBS1// |
| GO:1900180 | regulation of protein localization to nucleus | Biological process | 4 | 102 | 143 | 17653 | 4.84108048813931 | 0.00951046148482856 | 0.663085062081398 | 2.02179840888258 | 0.027972027972028 | SMO//ZC3H12A//KAT7//NMD3// |
| GO:0006366 | transcription by RNA polymerase II | Biological process | 30 | 2405 | 143 | 17653 | 1.53988630911708 | 0.00978682973459286 | 0.668477316592246 | 2.00935796721093 | 0.20979020979021 | CUX2//NR6A1//MITF//DLL4//H2AFY2//PURB//RARB//SMO//NCOA1//WDHD1//FOXP4//ALX3//QRICH1//HIF3A//SLTM//CREB5//TAF11//CDK12//TMEM100//KAT7//EBF3//HOXA9//PAX7//RNF4//ALX4//ZC3H12A//FUBP3//PCK1//CUL2//YBX2// |
| GO:2001237 | negative regulation of extrinsic apoptotic signaling pathway | Biological process | 4 | 103 | 143 | 17653 | 4.79407970670107 | 0.00983383352204815 | 0.668477316592246 | 2.00727714819837 | 0.027972027972028 | FAS//GFRAL//GCLM//THBS1// |
| GO:1903039 | positive regulation of leukocyte cell-cell adhesion | Biological process | 6 | 224 | 143 | 17653 | 3.30663086913087 | 0.00989322775973599 | 0.668477316592246 | 2.00466199257447 | 0.041958041958042 | MAP3K8//CD274//HLA-DPB1//VNN1//CD209//TFRC// |
| GO:0097345 | mitochondrial outer membrane permeabilization | Biological process | 3 | 55 | 143 | 17653 | 6.73350286077559 | 0.0100426388428945 | 0.668477316592246 | 1.998152155286 | 0.020979020979021 | PPP1R13B//MUL1//BAK1// |
| GO:0055082 | cellular chemical homeostasis | Biological process | 13 | 773 | 143 | 17653 | 2.07609079148536 | 0.010104349292611 | 0.668477316592246 | 1.99549164926671 | 0.0909090909090909 | TMEM199//TFRC//NCOA4//ATP6V0E1//TRPC5//BAK1//TRPC3//SLC41A1//FKBP1A//F2RL2//SLC30A2//GCLM//PCK1// |
| GO:0098581 | detection of external biotic stimulus | Biological process | 2 | 19 | 143 | 17653 | 12.9944792050055 | 0.0101808610984772 | 0.668477316592246 | 1.99221548776534 | 0.013986013986014 | NOD1//PGLYRP4// |
| GO:0010508 | positive regulation of autophagy | Biological process | 4 | 105 | 143 | 17653 | 4.7027639027639 | 0.0105018237820296 | 0.682921483056406 | 1.97873527334036 | 0.027972027972028 | PRKAA2//NOD1//MUL1//ZC3H12A// |
| GO:0031328 | positive regulation of cellular biosynthetic process | Biological process | 24 | 1812 | 143 | 17653 | 1.63506691983513 | 0.0107020228067662 | 0.684630255321237 | 1.97053412784857 | 0.167832167832168 | NCOA1//LARP4B//THBS1//EBF3//MITF//RNF4//NCOA4//FUBP3//CREB5//NPPC//TIMP2//PRKAA2//KAT7//NR6A1//HOXA9//PAX7//CDK12//RARB//ALX4//HIF3A//SMO//ZC3H12A//PCK1//NMD3// |
| GO:0050801 | ion homeostasis | Biological process | 13 | 779 | 143 | 17653 | 2.06010036176917 | 0.0107305644039703 | 0.684630255321237 | 1.96937743450344 | 0.0909090909090909 | TMEM199//TFRC//NCOA4//ATP6V0E1//TRPC5//BAK1//TRPC3//SLC41A1//FKBP1A//F2RL2//GCLM//PRKAA2//SLC30A2// |
| GO:0046486 | glycerolipid metabolic process | Biological process | 9 | 450 | 143 | 17653 | 2.46895104895105 | 0.0111191282886933 | 0.694821657345704 | 1.95392925899944 | 0.0629370629370629 | ETNK2//PIP5K1A//DGKA//AGPAT4//PIP5K1C//MTM1//SYNJ1//MOGAT3//PCK1// |
| GO:0008610 | lipid biosynthetic process | Biological process | 12 | 697 | 143 | 17653 | 2.12535240942701 | 0.0111971505396488 | 0.694821657345704 | 1.95089248289716 | 0.0839160839160839 | PRKAA2//ETNK2//DGKA//AGPAT4//PIP5K1C//MTM1//PIP5K1A//SYNJ1//DHCR24//IDI2//MOGAT3//PCK1// |
| GO:0051817 | modification of morphology or physiology of other organism involved in symbiotic interaction | Biological process | 4 | 107 | 143 | 17653 | 4.61486177374028 | 0.0111985155479346 | 0.694821657345704 | 1.95083954269304 | 0.027972027972028 | CLEC4M//CD209//TAF11//ZC3H12A// |
| GO:0006357 | regulation of transcription by RNA polymerase II | Biological process | 28 | 2227 | 143 | 17653 | 1.5521021412355 | 0.0114525998464841 | 0.702847757314904 | 1.94109591325299 | 0.195804195804196 | CUX2//NR6A1//MITF//DLL4//H2AFY2//PURB//RARB//SMO//NCOA1//TMEM100//KAT7//EBF3//HOXA9//PAX7//CDK12//RNF4//ALX4//HIF3A//ZC3H12A//FUBP3//PCK1//CUL2//WDHD1//FOXP4//ALX3//QRICH1//SLTM//CREB5// |
| GO:0014823 | response to activity | Biological process | 3 | 58 | 143 | 17653 | 6.38521823004582 | 0.0116060708735063 | 0.702847757314904 | 1.93531478170543 | 0.020979020979021 | PRKAA2//GCLM//PCK1// |
| GO:0031325 | positive regulation of cellular metabolic process | Biological process | 36 | 3074 | 143 | 17653 | 1.4457097879349 | 0.0119492415290991 | 0.702847757314904 | 1.92265966042136 | 0.251748251748252 | MAP3K8//NOD1//THBS1//NCOA1//FAS//CDK2AP1//MUL1//BAK1//TRAF4//PRKAA2//ZC3H12A//RIPK3//TRPC5//LARP4B//EBF3//MITF//RNF4//NCOA4//FUBP3//CREB5//NPPC//PAX7//FKBP1A//RASSF2//TIMP2//TFRC//KAT7//NR6A1//HOXA9//CDK12//RARB//ALX4//HIF3A//SMO//PCK1//NMD3// |
| GO:0070482 | response to oxygen levels | Biological process | 8 | 378 | 143 | 17653 | 2.61264661264661 | 0.0119668390910565 | 0.702847757314904 | 1.92202054855737 | 0.0559440559440559 | LTA//NPPC//THBS1//TMEM199//HIF3A//CUL2//FAS//PCK1// |
| GO:0048771 | tissue remodeling | Biological process | 5 | 169 | 143 | 17653 | 3.65229445111102 | 0.0122106133668757 | 0.702847757314904 | 1.91326251990779 | 0.034965034965035 | DLL4//BAK1//TFRC//MITF//RASSF2// |
| GO:0019400 | alditol metabolic process | Biological process | 2 | 21 | 143 | 17653 | 11.7569097569098 | 0.0123714687556238 | 0.702847757314904 | 1.9075787373477 | 0.013986013986014 | MOGAT3//PCK1// |
| GO:0043496 | regulation of protein homodimerization activity | Biological process | 2 | 21 | 143 | 17653 | 11.7569097569098 | 0.0123714687556238 | 0.702847757314904 | 1.9075787373477 | 0.013986013986014 | TRAF4//BAK1// |
| GO:0060713 | labyrinthine layer morphogenesis | Biological process | 2 | 21 | 143 | 17653 | 11.7569097569098 | 0.0123714687556238 | 0.702847757314904 | 1.9075787373477 | 0.013986013986014 | ST14//NCOA1// |
| GO:1901222 | regulation of NIK/NF-kappaB signaling | Biological process | 3 | 60 | 143 | 17653 | 6.17237762237762 | 0.0127218373207047 | 0.702847757314904 | 1.89545016222372 | 0.020979020979021 | TRAF4//RASSF2//NOD1// |
| GO:0009891 | positive regulation of biosynthetic process | Biological process | 24 | 1841 | 143 | 17653 | 1.60931084125001 | 0.0128471351957227 | 0.702847757314904 | 1.89119370559329 | 0.167832167832168 | NCOA1//LARP4B//THBS1//EBF3//MITF//RNF4//NCOA4//FUBP3//CREB5//NPPC//TIMP2//PRKAA2//KAT7//NR6A1//HOXA9//PAX7//CDK12//RARB//ALX4//HIF3A//SMO//ZC3H12A//PCK1//NMD3// |
| GO:0045216 | cell-cell junction organization | Biological process | 6 | 238 | 143 | 17653 | 3.1121231709467 | 0.0130470374721266 | 0.702847757314904 | 1.88448809028501 | 0.041958041958042 | CDH24//GJB2//PIP5K1C//WDR1//PIP5K1A//THBS1// |
| GO:0006355 | regulation of transcription, DNA-templated | Biological process | 42 | 3745 | 143 | 17653 | 1.38445853212208 | 0.0130845252910776 | 0.702847757314904 | 1.88324202901992 | 0.293706293706294 | CUX2//NR6A1//MITF//DLL4//H2AFY2//PURB//RARB//SMO//NCOA1//WDHD1//FOXP4//ALX3//QRICH1//HIF3A//SLTM//CREB5//EBF3//RNF4//NCOA4//FUBP3//ZNF488//TMEM100//NOD1//ZC3H12A//KAT7//HOXA9//PAX7//CDK12//ALX4//RIPK3//PCK1//CUL2//ZNF597//ZNF320//KDM2A//AFF3//YBX2//PRKAA2//TAF11//ZNF329//LZTR1//ZNF251// |
| GO:0043901 | negative regulation of multi-organism process | Biological process | 5 | 172 | 143 | 17653 | 3.58859164091722 | 0.0130953256894582 | 0.702847757314904 | 1.88288369591186 | 0.034965034965035 | LTA//ZC3H12A//MUL1//NPPC//MAPKBP1// |
| GO:0042110 | T cell activation | Biological process | 9 | 463 | 143 | 17653 | 2.39962844930448 | 0.0131940805186323 | 0.702847757314904 | 1.8796208699076 | 0.0629370629370629 | DLL4//MAP3K8//CD274//HLA-DPB1//RIPK3//VNN1//FKBP1A//CD209//TFRC// |
| GO:1902110 | positive regulation of mitochondrial membrane permeability involved in apoptotic process | Biological process | 3 | 61 | 143 | 17653 | 6.07119110397799 | 0.01330195005784 | 0.702847757314904 | 1.87608468705706 | 0.020979020979021 | PPP1R13B//MUL1//BAK1// |
| GO:0003416 | endochondral bone growth | Biological process | 2 | 22 | 143 | 17653 | 11.2225047679593 | 0.0135370168186482 | 0.702847757314904 | 1.86847703151084 | 0.013986013986014 | RARB//NPPC// |
| GO:0007035 | vacuolar acidification | Biological process | 2 | 22 | 143 | 17653 | 11.2225047679593 | 0.0135370168186482 | 0.702847757314904 | 1.86847703151084 | 0.013986013986014 | TMEM199//ATP6V0E1// |
| GO:0031667 | response to nutrient levels | Biological process | 9 | 465 | 143 | 17653 | 2.38930746672682 | 0.0135372841143693 | 0.702847757314904 | 1.86846845621598 | 0.0629370629370629 | NCOA1//COX4I1//GCLM//LTA//PRKAA2//TRPC3//FAS//ZC3H12A//PCK1// |
| GO:0030003 | cellular cation homeostasis | Biological process | 11 | 631 | 143 | 17653 | 2.15201755455321 | 0.013715451948558 | 0.702847757314904 | 1.86278987704735 | 0.0769230769230769 | TMEM199//TFRC//NCOA4//ATP6V0E1//TRPC5//BAK1//TRPC3//SLC41A1//FKBP1A//F2RL2//SLC30A2// |
| GO:0046916 | cellular transition metal ion homeostasis | Biological process | 4 | 114 | 143 | 17653 | 4.33149306833517 | 0.0138690733361487 | 0.702847757314904 | 1.85795255539691 | 0.027972027972028 | TMEM199//TFRC//NCOA4//SLC30A2// |
| GO:0006875 | cellular metal ion homeostasis | Biological process | 10 | 550 | 143 | 17653 | 2.24450095359186 | 0.0141472744811825 | 0.702847757314904 | 1.84932722033723 | 0.0699300699300699 | TMEM199//TFRC//NCOA4//TRPC5//BAK1//TRPC3//SLC41A1//FKBP1A//F2RL2//SLC30A2// |
| GO:0051252 | regulation of RNA metabolic process | Biological process | 44 | 3984 | 143 | 17653 | 1.3633765832561 | 0.0141513517085005 | 0.702847757314904 | 1.84920207522763 | 0.307692307692308 | CUX2//NR6A1//MITF//DLL4//H2AFY2//PURB//RARB//SMO//NCOA1//KAT7//ZNF597//ZNF320//KDM2A//AFF3//YBX2//PRKAA2//TAF11//ZNF329//LZTR1//ZNF251//WDHD1//FOXP4//ALX3//QRICH1//HIF3A//SLTM//CREB5//EBF3//RNF4//NCOA4//FUBP3//ZNF488//TMEM100//NOD1//ZC3H12A//CDK12//HOXA9//PAX7//ALX4//RIPK3//PCK1//CUL2//NMD3//NOVA2// |
| GO:0071345 | cellular response to cytokine stimulus | Biological process | 16 | 1081 | 143 | 17653 | 1.82716081328477 | 0.0142324835654528 | 0.702847757314904 | 1.84671930900507 | 0.111888111888112 | FKBP1A//STX1A//FAS//LTA//PDE1B//HLA-DPB1//MUL1//NOD1//MAP3K8//PCK1//ZC3H12A//THBS1//GCLM//TFRC//LAPTM5//PTPN7// |
| GO:2001235 | positive regulation of apoptotic signaling pathway | Biological process | 5 | 176 | 143 | 17653 | 3.50703273998729 | 0.0143410455689434 | 0.702847757314904 | 1.84341918421431 | 0.034965034965035 | BAK1//FAS//PPP1R13B//THBS1//RIPK3// |
| GO:1903506 | regulation of nucleic acid-templated transcription | Biological process | 42 | 3766 | 143 | 17653 | 1.37673850313256 | 0.0143596720484324 | 0.702847757314904 | 1.84285547856047 | 0.293706293706294 | CUX2//NR6A1//MITF//DLL4//H2AFY2//PURB//RARB//SMO//NCOA1//KAT7//ZNF597//ZNF320//KDM2A//AFF3//YBX2//PRKAA2//TAF11//ZNF329//LZTR1//ZNF251//WDHD1//FOXP4//ALX3//QRICH1//HIF3A//SLTM//CREB5//EBF3//RNF4//NCOA4//FUBP3//ZNF488//TMEM100//NOD1//ZC3H12A//HOXA9//PAX7//CDK12//ALX4//RIPK3//PCK1//CUL2// |
| GO:0043900 | regulation of multi-organism process | Biological process | 8 | 391 | 143 | 17653 | 2.5257811242466 | 0.0144174002718069 | 0.702847757314904 | 1.84111304401304 | 0.0559440559440559 | ZC3H12A//LTA//TAF11//MUL1//PGLYRP4//NPPC//MAPKBP1//NOD1// |
| GO:0048545 | response to steroid hormone | Biological process | 8 | 391 | 143 | 17653 | 2.5257811242466 | 0.0144174002718069 | 0.702847757314904 | 1.84111304401304 | 0.0559440559440559 | NR6A1//RNF4//NCOA4//NCOA1//GJB2//THBS1//RARB//PCK1// |
| GO:1902686 | mitochondrial outer membrane permeabilization involved in programmed cell death | Biological process | 3 | 63 | 143 | 17653 | 5.87845487845488 | 0.0145068991771304 | 0.702847757314904 | 1.8384254072849 | 0.020979020979021 | PPP1R13B//MUL1//BAK1// |
| GO:0033993 | response to lipid | Biological process | 14 | 900 | 143 | 17653 | 1.92029526029526 | 0.0145191735168459 | 0.702847757314904 | 1.8380581045236 | 0.0979020979020979 | NR6A1//RNF4//NCOA4//NCOA1//GJB2//FAS//LTA//PCK1//RARB//THBS1//CD274//ZC3H12A//PRKAA2//SMO// |
| GO:0021513 | spinal cord dorsal/ventral patterning | Biological process | 2 | 23 | 143 | 17653 | 10.734569778048 | 0.0147483020918562 | 0.702847757314904 | 1.83125797525073 | 0.013986013986014 | SMO//DLL4// |
| GO:0021904 | dorsal/ventral neural tube patterning | Biological process | 2 | 23 | 143 | 17653 | 10.734569778048 | 0.0147483020918562 | 0.702847757314904 | 1.83125797525073 | 0.013986013986014 | PAX7//SMO// |
| GO:0042744 | hydrogen peroxide catabolic process | Biological process | 2 | 23 | 143 | 17653 | 10.734569778048 | 0.0147483020918562 | 0.702847757314904 | 1.83125797525073 | 0.013986013986014 | GPX3//PRDX2// |
| GO:0030099 | myeloid cell differentiation | Biological process | 8 | 394 | 143 | 17653 | 2.50654928827518 | 0.0150313918743657 | 0.702847757314904 | 1.82300080282169 | 0.0559440559440559 | WDR1//PDE1B//MITF//TFRC//PURB//HOXA9//THBS1//RASSF2// |
| GO:0051881 | regulation of mitochondrial membrane potential | Biological process | 3 | 64 | 143 | 17653 | 5.78660402097902 | 0.0151318282764545 | 0.702847757314904 | 1.82010859594203 | 0.020979020979021 | GCLM//BAK1//MUL1// |
| GO:1903555 | regulation of tumor necrosis factor superfamily cytokine production | Biological process | 4 | 118 | 143 | 17653 | 4.18466279483229 | 0.0155619085965789 | 0.702847757314904 | 1.80793713985723 | 0.027972027972028 | ZC3H12A//NOD1//THBS1//CD274// |
| GO:0006879 | cellular iron ion homeostasis | Biological process | 3 | 65 | 143 | 17653 | 5.69757934373319 | 0.015771781787229 | 0.702847757314904 | 1.80211924030145 | 0.020979020979021 | TMEM199//TFRC//NCOA4// |
| GO:0035794 | positive regulation of mitochondrial membrane permeability | Biological process | 3 | 65 | 143 | 17653 | 5.69757934373319 | 0.015771781787229 | 0.702847757314904 | 1.80211924030145 | 0.020979020979021 | PPP1R13B//MUL1//BAK1// |
| GO:0050863 | regulation of T cell activation | Biological process | 7 | 321 | 143 | 17653 | 2.6920027013485 | 0.0157753800449099 | 0.702847757314904 | 1.80202016936312 | 0.048951048951049 | MAP3K8//CD274//HLA-DPB1//VNN1//CD209//TFRC//RIPK3// |
| GO:0006873 | cellular ion homeostasis | Biological process | 11 | 645 | 143 | 17653 | 2.10530709600477 | 0.0159041736979746 | 0.702847757314904 | 1.79848888975889 | 0.0769230769230769 | TMEM199//TFRC//NCOA4//ATP6V0E1//TRPC5//BAK1//TRPC3//SLC41A1//FKBP1A//F2RL2//SLC30A2// |
| GO:0050767 | regulation of neurogenesis | Biological process | 12 | 732 | 143 | 17653 | 2.02373036799266 | 0.0159423452046527 | 0.702847757314904 | 1.79744779118822 | 0.0839160839160839 | SMO//PMP22//RARB//TIMP2//NCOA1//TRPC5//WDR1//ZNF488//CUX2//LTA//MUL1//DLL4// |
| GO:2000116 | regulation of cysteine-type endopeptidase activity | Biological process | 6 | 249 | 143 | 17653 | 2.97463981801331 | 0.0159759407751143 | 0.702847757314904 | 1.79653355812037 | 0.041958041958042 | NOD1//FAS//MUL1//BAK1//DHCR24//THBS1// |
| GO:0002068 | glandular epithelial cell development | Biological process | 2 | 24 | 143 | 17653 | 10.287296037296 | 0.0160045179101723 | 0.702847757314904 | 1.7957574031884 | 0.013986013986014 | SMO//RARB// |
| GO:0008053 | mitochondrial fusion | Biological process | 2 | 24 | 143 | 17653 | 10.287296037296 | 0.0160045179101723 | 0.702847757314904 | 1.7957574031884 | 0.013986013986014 | MUL1//BAK1// |
| GO:0033233 | regulation of protein sumoylation | Biological process | 2 | 24 | 143 | 17653 | 10.287296037296 | 0.0160045179101723 | 0.702847757314904 | 1.7957574031884 | 0.013986013986014 | RNF4//MUL1// |
| GO:0098868 | bone growth | Biological process | 2 | 24 | 143 | 17653 | 10.287296037296 | 0.0160045179101723 | 0.702847757314904 | 1.7957574031884 | 0.013986013986014 | RARB//NPPC// |
| GO:1901654 | response to ketone | Biological process | 5 | 183 | 143 | 17653 | 3.37288394665444 | 0.0167083361047952 | 0.70501698198327 | 1.77706679705369 | 0.034965034965035 | GJB2//THBS1//PRKAA2//PCK1//KIF1B// |
| GO:0019637 | organophosphate metabolic process | Biological process | 17 | 1196 | 143 | 17653 | 1.75468929064247 | 0.0167275261328838 | 0.70501698198327 | 1.7765682829635 | 0.118881118881119 | H6PD//COX4I1//NPPC//PDE1B//ETNK2//PIP5K1A//DGKA//AGPAT4//PIP5K1C//MTM1//SYNJ1//ACPP//IDI2//MCCC2//THBS1//TIMP2//PRKAA2// |
| GO:0006695 | cholesterol biosynthetic process | Biological process | 3 | 67 | 143 | 17653 | 5.52750234839787 | 0.0170969013720824 | 0.70501698198327 | 1.76708259363984 | 0.020979020979021 | DHCR24//PRKAA2//IDI2// |
| GO:0030521 | androgen receptor signaling pathway | Biological process | 3 | 67 | 143 | 17653 | 5.52750234839787 | 0.0170969013720824 | 0.70501698198327 | 1.76708259363984 | 0.020979020979021 | RNF4//NCOA4//NCOA1// |
| GO:1902108 | regulation of mitochondrial membrane permeability involved in apoptotic process | Biological process | 3 | 67 | 143 | 17653 | 5.52750234839787 | 0.0170969013720824 | 0.70501698198327 | 1.76708259363984 | 0.020979020979021 | PPP1R13B//MUL1//BAK1// |
| GO:1905710 | positive regulation of membrane permeability | Biological process | 3 | 67 | 143 | 17653 | 5.52750234839787 | 0.0170969013720824 | 0.70501698198327 | 1.76708259363984 | 0.020979020979021 | PPP1R13B//MUL1//BAK1// |
| GO:0021511 | spinal cord patterning | Biological process | 2 | 25 | 143 | 17653 | 9.8758041958042 | 0.0173048675157804 | 0.70501698198327 | 1.76183172126935 | 0.013986013986014 | SMO//DLL4// |
| GO:0046856 | phosphatidylinositol dephosphorylation | Biological process | 2 | 25 | 143 | 17653 | 9.8758041958042 | 0.0173048675157804 | 0.70501698198327 | 1.76183172126935 | 0.013986013986014 | MTM1//SYNJ1// |
| GO:0060351 | cartilage development involved in endochondral bone morphogenesis | Biological process | 2 | 25 | 143 | 17653 | 9.8758041958042 | 0.0173048675157804 | 0.70501698198327 | 1.76183172126935 | 0.013986013986014 | RARB//NPPC// |
| GO:0060669 | embryonic placenta morphogenesis | Biological process | 2 | 25 | 143 | 17653 | 9.8758041958042 | 0.0173048675157804 | 0.70501698198327 | 1.76183172126935 | 0.013986013986014 | ST14//NCOA1// |
| GO:1903588 | negative regulation of blood vessel endothelial cell proliferation involved in sprouting angiogenesis | Biological process | 2 | 25 | 143 | 17653 | 9.8758041958042 | 0.0173048675157804 | 0.70501698198327 | 1.76183172126935 | 0.013986013986014 | DLL4//THBS1// |
| GO:2000463 | positive regulation of excitatory postsynaptic potential | Biological process | 2 | 25 | 143 | 17653 | 9.8758041958042 | 0.0173048675157804 | 0.70501698198327 | 1.76183172126935 | 0.013986013986014 | CUX2//STX1A// |
| GO:0030810 | positive regulation of nucleotide biosynthetic process | Biological process | 3 | 68 | 143 | 17653 | 5.44621554915673 | 0.0177821270562547 | 0.705519705775521 | 1.75001629097309 | 0.020979020979021 | NPPC//TIMP2//PRKAA2// |
| GO:1900373 | positive regulation of purine nucleotide biosynthetic process | Biological process | 3 | 68 | 143 | 17653 | 5.44621554915673 | 0.0177821270562547 | 0.705519705775521 | 1.75001629097309 | 0.020979020979021 | NPPC//TIMP2//PRKAA2// |
| GO:0048706 | embryonic skeletal system development | Biological process | 4 | 123 | 143 | 17653 | 4.01455455113992 | 0.0178538664630742 | 0.705519705775521 | 1.7482677178296 | 0.027972027972028 | ALX3//ALX4//HOXA9//PAX7// |
| GO:0071706 | tumor necrosis factor superfamily cytokine production | Biological process | 4 | 123 | 143 | 17653 | 4.01455455113992 | 0.0178538664630742 | 0.705519705775521 | 1.7482677178296 | 0.027972027972028 | ZC3H12A//NOD1//THBS1//CD274// |
| GO:0007249 | I-kappaB kinase/NF-kappaB signaling | Biological process | 6 | 256 | 143 | 17653 | 2.89330201048951 | 0.018060560128312 | 0.705519705775521 | 1.74326878465027 | 0.041958041958042 | NOD1//FKBP1A//MUL1//MAPKBP1//ZC3H12A//RIPK3// |
| GO:0045597 | positive regulation of cell differentiation | Biological process | 14 | 926 | 143 | 17653 | 1.86637768279237 | 0.0181449874234104 | 0.705519705775521 | 1.74124332847998 | 0.0979020979020979 | SMO//VNN1//ZC3H12A//TMEM100//H2AFY2//RARB//TIMP2//NCOA1//NPPC//TRPC5//ZNF488//CUX2//LTA//MUL1// |
| GO:0003206 | cardiac chamber morphogenesis | Biological process | 4 | 124 | 143 | 17653 | 3.98217911121137 | 0.0183361119795273 | 0.705519705775521 | 1.73669274744709 | 0.027972027972028 | RARB//DLL4//FKBP1A//SMO// |
| GO:0046330 | positive regulation of JNK cascade | Biological process | 4 | 124 | 143 | 17653 | 3.98217911121137 | 0.0183361119795273 | 0.705519705775521 | 1.73669274744709 | 0.027972027972028 | MUL1//NOD1//TRAF4//RASSF2// |
| GO:0022409 | positive regulation of cell-cell adhesion | Biological process | 6 | 257 | 143 | 17653 | 2.8820440260129 | 0.0183728860707236 | 0.705519705775521 | 1.7358226179925 | 0.041958041958042 | MAP3K8//CD274//HLA-DPB1//VNN1//CD209//TFRC// |
| GO:1902653 | secondary alcohol biosynthetic process | Biological process | 3 | 69 | 143 | 17653 | 5.36728488902402 | 0.0184824965344655 | 0.705519705775521 | 1.73323936656221 | 0.020979020979021 | DHCR24//PRKAA2//IDI2// |
| GO:0033137 | negative regulation of peptidyl-serine phosphorylation | Biological process | 2 | 26 | 143 | 17653 | 9.49596557288865 | 0.0186485639523957 | 0.705519705775521 | 1.72935460576214 | 0.013986013986014 | BAK1//RASSF2// |
| GO:0032774 | RNA biosynthetic process | Biological process | 43 | 3940 | 143 | 17653 | 1.34727024244791 | 0.0187990506951942 | 0.705519705775521 | 1.72586408096228 | 0.300699300699301 | CUX2//NR6A1//MITF//DLL4//H2AFY2//PURB//RARB//SMO//NCOA1//EBF3//YBX2//ALX4//HIF3A//TAF11//FUBP3//CREB5//KAT7//FOXP4//ZNF488//ZNF597//ZNF320//KDM2A//ALX3//HOXA9//AFF3//PAX7//PRKAA2//RNF4//ZNF329//SLTM//ZC3H12A//NCOA4//ZNF251//LZTR1//WDHD1//QRICH1//CDK12//TMEM100//NOD1//RIPK3//PCK1//CUL2//NMD3// |
| GO:0008637 | apoptotic mitochondrial changes | Biological process | 4 | 125 | 143 | 17653 | 3.95032167832168 | 0.0188263951145158 | 0.705519705775521 | 1.7252328308996 | 0.027972027972028 | BAK1//PPP1R13B//MUL1//GCLM// |
| GO:0051171 | regulation of nitrogen compound metabolic process | Biological process | 63 | 6240 | 143 | 17653 | 1.24634548144164 | 0.0189631625632944 | 0.705519705775521 | 1.72208923191091 | 0.440559440559441 | CUX2//NR6A1//MITF//DLL4//H2AFY2//PURB//RARB//SMO//MAP3K8//NOD1//THBS1//NCOA1//FKBP1A//ZC3H12A//FAS//CDK2AP1//KAT7//ZNF597//ZNF320//KDM2A//AFF3//YBX2//PRKAA2//TAF11//ZNF329//LZTR1//ZNF251//WDHD1//FOXP4//ALX3//QRICH1//HIF3A//SLTM//CREB5//MUL1//BAK1//TRAF4//USP51//TRPC5//TIMP2//LARP4B//EBF3//RNF4//NCOA4//FUBP3//ZNF488//TMEM100//NPPC//PAX7//RASSF2//RIPK3//MTM1//PDE1B//NECAB3//DHCR24//CDK12//TFRC//HOXA9//ALX4//NOVA2//PCK1//CUL2//NMD3// |
| GO:0051962 | positive regulation of nervous system development | Biological process | 9 | 493 | 143 | 17653 | 2.25360643413382 | 0.0190682445480275 | 0.705519705775521 | 1.71968928693286 | 0.0629370629370629 | SMO//RARB//TIMP2//NCOA1//TRPC5//ZNF488//CUX2//LTA//MUL1// |
| GO:0051240 | positive regulation of multicellular organismal process | Biological process | 21 | 1606 | 143 | 17653 | 1.61419589128182 | 0.0191875520452257 | 0.705519705775521 | 1.7169804291762 | 0.146853146853147 | SMO//THBS1//ZC3H12A//HLA-DPB1//LTA//NOD1//VNN1//H2AFY2//RARB//TIMP2//NCOA1//NPPC//TRPC5//TFRC//MTM1//ZNF488//CUX2//TRPC3//MUL1//CD274//TMEM100// |
| GO:0070227 | lymphocyte apoptotic process | Biological process | 3 | 70 | 143 | 17653 | 5.29060939060939 | 0.0191980299207153 | 0.705519705775521 | 1.71674333579962 | 0.020979020979021 | BAK1//RIPK3//CD274// |
| GO:0038061 | NIK/NF-kappaB signaling | Biological process | 4 | 126 | 143 | 17653 | 3.91896991896992 | 0.0193247507568647 | 0.705519705775521 | 1.71388609873519 | 0.027972027972028 | TRAF4//RASSF2//NOD1//RIPK3// |
| GO:0046651 | lymphocyte proliferation | Biological process | 6 | 260 | 143 | 17653 | 2.84878967186659 | 0.0193320697772307 | 0.705519705775521 | 1.71372164598652 | 0.041958041958042 | TFRC//FKBP1A//CD274//CD209//HLA-DPB1//RIPK3// |
| GO:0007159 | leukocyte cell-cell adhesion | Biological process | 7 | 335 | 143 | 17653 | 2.57950109591901 | 0.0194513690727071 | 0.705519705775521 | 1.71104982571118 | 0.048951048951049 | MAP3K8//CD274//HLA-DPB1//VNN1//CD209//TFRC//CLEC4M// |
| GO:0009991 | response to extracellular stimulus | Biological process | 9 | 495 | 143 | 17653 | 2.24450095359186 | 0.0195180306675123 | 0.705519705775521 | 1.70956400395307 | 0.0629370629370629 | NCOA1//COX4I1//GCLM//LTA//PRKAA2//TRPC3//FAS//ZC3H12A//PCK1// |
| GO:0061024 | membrane organization | Biological process | 14 | 936 | 143 | 17653 | 1.8464377502839 | 0.0197104591207988 | 0.705519705775521 | 1.70530325946956 | 0.0979020979020979 | STX1A//TMEM170A//LYZL6//BAK1//MUL1//COX18//THBS1//PPP1R13B//SHISA6//DHCR24//PIP5K1C//TFRC//CLTCL1//SYNJ1// |
| GO:0043401 | steroid hormone mediated signaling pathway | Biological process | 5 | 191 | 143 | 17653 | 3.23161132061656 | 0.0197165790908729 | 0.705519705775521 | 1.70516843477186 | 0.034965034965035 | NR6A1//RNF4//NCOA4//NCOA1//RARB// |
| GO:0002831 | regulation of response to biotic stimulus | Biological process | 4 | 127 | 143 | 17653 | 3.88811188811189 | 0.0198312127481686 | 0.705886799030864 | 1.7026507263528 | 0.027972027972028 | ZC3H12A//MUL1//MAPKBP1//NOD1// |
| GO:0032943 | mononuclear cell proliferation | Biological process | 6 | 262 | 143 | 17653 | 2.82704318582181 | 0.0199902166988898 | 0.70782112845336 | 1.69918249799684 | 0.041958041958042 | TFRC//FKBP1A//CD274//CD209//HLA-DPB1//RIPK3// |
| GO:0003006 | developmental process involved in reproduction | Biological process | 11 | 669 | 143 | 17653 | 2.02978038404047 | 0.0202554835274196 | 0.713478307791348 | 1.69345738512499 | 0.0769230769230769 | ETNK2//DZIP1//HOXA9//NCOA4//DHCR24//GJB2//YBX2//BAK1//ST14//NCOA1//NPPC// |
| GO:0034332 | adherens junction organization | Biological process | 4 | 129 | 143 | 17653 | 3.82783108364504 | 0.0208685859176221 | 0.724531025242249 | 1.68050697829345 | 0.027972027972028 | PIP5K1C//PIP5K1A//THBS1//CDH24// |
| GO:0043280 | positive regulation of cysteine-type endopeptidase activity involved in apoptotic process | Biological process | 4 | 129 | 143 | 17653 | 3.82783108364504 | 0.0208685859176221 | 0.724531025242249 | 1.68050697829345 | 0.027972027972028 | NOD1//FAS//MUL1//BAK1// |
| GO:0071310 | cellular response to organic substance | Biological process | 31 | 2656 | 143 | 17653 | 1.4408411618502 | 0.0210087424320797 | 0.724531025242249 | 1.67759994336531 | 0.216783216783217 | NCOA1//BAK1//FKBP1A//ATP6V0E1//RARB//STX1A//TMEM100//THBS1//NR6A1//RNF4//NCOA4//PCK1//FAS//LTA//GCLM//DLL4//PDE1B//HLA-DPB1//MUL1//NOD1//MAP3K8//CD274//ZC3H12A//PTPN7//GJB2//PRKAA2//SMO//KIF1B//TFRC//LAPTM5//CUX2// |
| GO:0045944 | positive regulation of transcription by RNA polymerase II | Biological process | 16 | 1132 | 143 | 17653 | 1.74484173070746 | 0.0210389082696472 | 0.724531025242249 | 1.67697679991608 | 0.111888111888112 | NCOA1//PCK1//KAT7//EBF3//NR6A1//HOXA9//MITF//PAX7//CDK12//RARB//RNF4//ALX4//HIF3A//SMO//ZC3H12A//FUBP3// |
| GO:0035556 | intracellular signal transduction | Biological process | 33 | 2871 | 143 | 17653 | 1.41893738445463 | 0.0211049256206895 | 0.724531025242249 | 1.67561617407489 | 0.230769230769231 | MAP3K8//NOD1//THBS1//SMO//RIPK3//TRAF4//MUL1//DHCR24//BAK1//PIP5K1C//PIP5K1A//KAT7//GFRAL//MTM1//PRKAA2//TIMP2//FAS//F2RL2//FKBP1A//MAPKBP1//ZC3H12A//CDK12//TMEM100//RASSF2//PPP1R13B//CUL2//TAF11//VNN1//CLEC4M//DGKA//CD209//CAMK1G//RGS7// |
| GO:0030823 | regulation of cGMP metabolic process | Biological process | 2 | 28 | 143 | 17653 | 8.81768231768232 | 0.0214628978740964 | 0.731687423199992 | 1.66831164090858 | 0.013986013986014 | NPPC//THBS1// |
| GO:0009607 | response to biotic stimulus | Biological process | 14 | 947 | 143 | 17653 | 1.82499021569771 | 0.0215490269933772 | 0.731687423199992 | 1.66657233482795 | 0.0979020979020979 | ZC3H12A//NOD1//LYZL6//LTA//PGLYRP4//BAK1//GJB2//FAS//PCK1//MUL1//SMO//CD274//MAPKBP1//PRRT1// |
| GO:0010720 | positive regulation of cell development | Biological process | 9 | 504 | 143 | 17653 | 2.20442057942058 | 0.0216379542569863 | 0.731687423199992 | 1.66478380164773 | 0.0629370629370629 | SMO//RARB//TIMP2//NCOA1//TRPC5//ZNF488//CUX2//LTA//MUL1// |
| GO:0060284 | regulation of cell development | Biological process | 13 | 858 | 143 | 17653 | 1.87041746132655 | 0.022125148759191 | 0.738125161653108 | 1.65511380062658 | 0.0909090909090909 | SMO//PMP22//RARB//TIMP2//NCOA1//TRPC5//WDR1//ZNF488//DLL4//CUX2//LTA//NPPC//MUL1// |
| GO:0016126 | sterol biosynthetic process | Biological process | 3 | 74 | 143 | 17653 | 5.0046305046305 | 0.0222120757350883 | 0.738125161653108 | 1.65341085441094 | 0.020979020979021 | DHCR24//PRKAA2//IDI2// |
| GO:0007275 | multicellular organism development | Biological process | 55 | 5351 | 143 | 17653 | 1.268849819588 | 0.0224212978673455 | 0.738125161653108 | 1.64933925167359 | 0.384615384615385 | ALX4//RASSF2//MCAM//DLL4//TMEM100//HIF3A//ZC3H12A//SMO//RARB//ETNK2//ST14//THBS1//BAK1//FKBP1A//NPPC//ALX3//GFRAL//TRPC5//TIMP2//H2AFY2//PMP22//RIPK3//LTA//HOXA9//NCOA4//ZNF488//PAX7//TACC1//PIP5K1A//WDR1//PDE1B//MITF//TFRC//TRAF4//DHCR24//KRT6C//KRTAP4-4//VNN1//AFF3//TPRA1//PURB//NCOA1//GJB2//MTM1//CUX2//TCHH//PCK1//MUL1//EDIL3//DZIP1//EBF3//FAS//QRICH1//CDH24//KCTD15// |
| GO:0007006 | mitochondrial membrane organization | Biological process | 4 | 132 | 143 | 17653 | 3.7408349226531 | 0.022486229308503 | 0.738125161653108 | 1.64808336483139 | 0.027972027972028 | COX18//BAK1//PPP1R13B//MUL1// |
| GO:0050671 | positive regulation of lymphocyte proliferation | Biological process | 4 | 132 | 143 | 17653 | 3.7408349226531 | 0.022486229308503 | 0.738125161653108 | 1.64808336483139 | 0.027972027972028 | TFRC//CD274//CD209//HLA-DPB1// |
| GO:0009617 | response to bacterium | Biological process | 10 | 593 | 143 | 17653 | 2.08174624700763 | 0.0225923271421253 | 0.738125161653108 | 1.64603903189816 | 0.0699300699300699 | NOD1//LYZL6//LTA//PGLYRP4//GJB2//FAS//PCK1//CD274//ZC3H12A//MAPKBP1// |
| GO:0097190 | apoptotic signaling pathway | Biological process | 10 | 593 | 143 | 17653 | 2.08174624700763 | 0.0225923271421253 | 0.738125161653108 | 1.64603903189816 | 0.0699300699300699 | BAK1//PPP1R13B//FAS//CUL2//MUL1//THBS1//VNN1//GCLM//GFRAL//RIPK3// |
| GO:0034698 | response to gonadotropin | Biological process | 2 | 29 | 143 | 17653 | 8.51362430672775 | 0.0229320095172745 | 0.745612066558681 | 1.63955788665582 | 0.013986013986014 | GJB2//GCLM// |
| GO:0032946 | positive regulation of mononuclear cell proliferation | Biological process | 4 | 133 | 143 | 17653 | 3.71270834428729 | 0.0230419816517469 | 0.745612066558681 | 1.63748017353831 | 0.027972027972028 | TFRC//CD274//CD209//HLA-DPB1// |
| GO:0071229 | cellular response to acid chemical | Biological process | 5 | 200 | 143 | 17653 | 3.08618881118881 | 0.0235028783604526 | 0.754782178572382 | 1.62887894711072 | 0.034965034965035 | PCK1//RARB//PRKAA2//GCLM//ZC3H12A// |
| GO:0042737 | drug catabolic process | Biological process | 4 | 134 | 143 | 17653 | 3.68500156559858 | 0.0236060480754226 | 0.754782178572382 | 1.62697671273885 | 0.027972027972028 | PCK1//GPX3//PRDX2//SATL1// |
| GO:0048856 | anatomical structure development | Biological process | 59 | 5836 | 143 | 17653 | 1.24801329582001 | 0.0239447199807592 | 0.754782178572382 | 1.62079023740702 | 0.412587412587413 | CDH24//ALX4//RASSF2//MCAM//DLL4//TMEM100//HIF3A//ZC3H12A//SMO//RARB//ETNK2//ST14//THBS1//BAK1//FKBP1A//NPPC//EDIL3//DZIP1//CUX2//EBF3//HOXA9//FAS//QRICH1//KCTD15//FOXP4//PAX7//LZTR1//CLTCL1//ALX3//DHCR24//GFRAL//TRPC5//TIMP2//H2AFY2//PMP22//RIPK3//LTA//WDR1//NCOA4//MUL1//ZNF488//TACC1//PIP5K1A//PDE1B//MITF//TFRC//TRAF4//KRT6C//KRTAP4-4//VNN1//AFF3//TPRA1//PURB//NCOA1//GJB2//YBX2//MTM1//TCHH//PCK1// |
| GO:0050670 | regulation of lymphocyte proliferation | Biological process | 5 | 201 | 143 | 17653 | 3.07083463799882 | 0.0239505675176412 | 0.754782178572382 | 1.62068419135757 | 0.034965034965035 | TFRC//CD274//CD209//HLA-DPB1//RIPK3// |
| GO:0048608 | reproductive structure development | Biological process | 8 | 430 | 143 | 17653 | 2.29669865018702 | 0.0239546094578387 | 0.754782178572382 | 1.62061090523532 | 0.0559440559440559 | ETNK2//HOXA9//NCOA4//DHCR24//GJB2//BAK1//ST14//NCOA1// |
| GO:0002696 | positive regulation of leukocyte activation | Biological process | 7 | 351 | 143 | 17653 | 2.46191700037854 | 0.0243444870207068 | 0.754782178572382 | 1.61359937233696 | 0.048951048951049 | TFRC//MAP3K8//CD274//HLA-DPB1//VNN1//CD209//THBS1// |
| GO:0032944 | regulation of mononuclear cell proliferation | Biological process | 5 | 202 | 143 | 17653 | 3.05563248632556 | 0.02440373487198 | 0.754782178572382 | 1.61254370193328 | 0.034965034965035 | TFRC//CD274//CD209//HLA-DPB1//RIPK3// |
| GO:0032689 | negative regulation of interferon-gamma production | Biological process | 2 | 30 | 143 | 17653 | 8.22983682983683 | 0.0244414161034085 | 0.754782178572382 | 1.61187363525111 | 0.013986013986014 | ZC3H12A//CD274// |
| GO:0036296 | response to increased oxygen levels | Biological process | 2 | 30 | 143 | 17653 | 8.22983682983683 | 0.0244414161034085 | 0.754782178572382 | 1.61187363525111 | 0.013986013986014 | TMEM199//FAS// |
| GO:0046685 | response to arsenic-containing substance | Biological process | 2 | 30 | 143 | 17653 | 8.22983682983683 | 0.0244414161034085 | 0.754782178572382 | 1.61187363525111 | 0.013986013986014 | ZC3H12A//RNF4// |
| GO:0031295 | T cell costimulation | Biological process | 3 | 77 | 143 | 17653 | 4.80964490055399 | 0.0246322776090733 | 0.757218606682558 | 1.6084954295202 | 0.020979020979021 | MAP3K8//CD274//HLA-DPB1// |
| GO:0061458 | reproductive system development | Biological process | 8 | 433 | 143 | 17653 | 2.28078618840744 | 0.0248366323344272 | 0.758840028400589 | 1.60490729165676 | 0.0559440559440559 | ETNK2//HOXA9//NCOA4//DHCR24//GJB2//BAK1//ST14//NCOA1// |
| GO:1902680 | positive regulation of RNA biosynthetic process | Biological process | 19 | 1451 | 143 | 17653 | 1.61647380875499 | 0.0252402461727023 | 0.758840028400589 | 1.59790641365434 | 0.132867132867133 | NCOA1//EBF3//MITF//RNF4//NCOA4//FUBP3//CREB5//KAT7//NR6A1//HOXA9//PAX7//CDK12//RARB//ALX4//HIF3A//SMO//ZC3H12A//PCK1//NMD3// |
| GO:0031294 | lymphocyte costimulation | Biological process | 3 | 78 | 143 | 17653 | 4.74798278644433 | 0.025469414154446 | 0.758840028400589 | 1.59398104453059 | 0.020979020979021 | MAP3K8//CD274//HLA-DPB1// |
| GO:0006979 | response to oxidative stress | Biological process | 8 | 436 | 143 | 17653 | 2.26509270545968 | 0.0257412397039181 | 0.758840028400589 | 1.58937054120762 | 0.0559440559440559 | GPX3//PRDX2//GJB2//ZC3H12A//BAK1//VNN1//DHCR24//GCLM// |
| GO:0070661 | leukocyte proliferation | Biological process | 6 | 278 | 143 | 17653 | 2.66433566433566 | 0.0258115248700755 | 0.758840028400589 | 1.58818633783903 | 0.041958041958042 | TFRC//FKBP1A//CD274//CD209//HLA-DPB1//RIPK3// |
| GO:0006351 | transcription, DNA-templated | Biological process | 42 | 3907 | 143 | 17653 | 1.32705328968446 | 0.0258445787381888 | 0.758840028400589 | 1.58763054235762 | 0.293706293706294 | CUX2//NR6A1//MITF//DLL4//H2AFY2//PURB//RARB//SMO//NCOA1//EBF3//YBX2//ALX4//HIF3A//TAF11//FUBP3//CREB5//KAT7//ZNF597//ZNF320//KDM2A//AFF3//PRKAA2//ZNF329//LZTR1//ZNF251//WDHD1//FOXP4//ALX3//QRICH1//SLTM//CDK12//RNF4//NCOA4//ZNF488//TMEM100//NOD1//ZC3H12A//HOXA9//PAX7//RIPK3//PCK1//CUL2// |
| GO:0030518 | intracellular steroid hormone receptor signaling pathway | Biological process | 4 | 138 | 143 | 17653 | 3.57818992601601 | 0.0259459500296306 | 0.758840028400589 | 1.58593042267295 | 0.027972027972028 | RNF4//NCOA4//NCOA1//NR6A1// |
| GO:0003209 | cardiac atrium morphogenesis | Biological process | 2 | 31 | 143 | 17653 | 7.96435822242274 | 0.0259903781341479 | 0.758840028400589 | 1.58518740190033 | 0.013986013986014 | SMO//DLL4// |
| GO:0042307 | positive regulation of protein import into nucleus | Biological process | 2 | 31 | 143 | 17653 | 7.96435822242274 | 0.0259903781341479 | 0.758840028400589 | 1.58518740190033 | 0.013986013986014 | SMO//ZC3H12A// |
| GO:0043393 | regulation of protein binding | Biological process | 5 | 206 | 143 | 17653 | 2.99629981668817 | 0.0262715946512673 | 0.758840028400589 | 1.58051356530958 | 0.034965034965035 | FKBP1A//NMD3//BAK1//TRAF4//PAX7// |
| GO:0031323 | regulation of cellular metabolic process | Biological process | 64 | 6454 | 143 | 17653 | 1.22414678596891 | 0.0263078418712366 | 0.758840028400589 | 1.57991477722238 | 0.447552447552448 | CUX2//NR6A1//MITF//DLL4//H2AFY2//PURB//RARB//SMO//MAP3K8//NOD1//THBS1//NCOA1//FKBP1A//ZC3H12A//FAS//CDK2AP1//KAT7//ZNF597//ZNF320//KDM2A//AFF3//YBX2//PRKAA2//TAF11//ZNF329//LZTR1//ZNF251//WDHD1//FOXP4//ALX3//QRICH1//HIF3A//SLTM//CREB5//MUL1//BAK1//TRAF4//USP51//RIPK3//TRPC5//TIMP2//LARP4B//ATP6V0E1//EBF3//RNF4//NCOA4//FUBP3//ZNF488//TMEM100//NPPC//PAX7//RASSF2//MTM1//PDE1B//NECAB3//DHCR24//CDK12//TFRC//HOXA9//ALX4//NOVA2//PCK1//CUL2//NMD3// |
| GO:0046902 | regulation of mitochondrial membrane permeability | Biological process | 3 | 79 | 143 | 17653 | 4.68788173851465 | 0.0263217366087012 | 0.758840028400589 | 1.57968546100428 | 0.020979020979021 | PPP1R13B//MUL1//BAK1// |
| GO:0009267 | cellular response to starvation | Biological process | 4 | 139 | 143 | 17653 | 3.55244755244755 | 0.0265519495179587 | 0.758840028400589 | 1.57590358630346 | 0.027972027972028 | FAS//PRKAA2//ZC3H12A//PCK1// |
| GO:0007584 | response to nutrient | Biological process | 5 | 207 | 143 | 17653 | 2.98182493834668 | 0.0267524557581381 | 0.758840028400589 | 1.57263634548366 | 0.034965034965035 | NCOA1//TRPC3//COX4I1//GCLM//LTA// |
| GO:0048534 | hematopoietic or lymphoid organ development | Biological process | 13 | 882 | 143 | 17653 | 1.81952174809318 | 0.0269413180700722 | 0.758840028400589 | 1.56958116078244 | 0.0909090909090909 | BAK1//RIPK3//LTA//DLL4//WDR1//PDE1B//MITF//TFRC//VNN1//PURB//HOXA9//THBS1//RASSF2// |
| GO:0090407 | organophosphate biosynthetic process | Biological process | 11 | 700 | 143 | 17653 | 1.93989010989011 | 0.0271119512986106 | 0.758840028400589 | 1.56683922429622 | 0.0769230769230769 | NPPC//ETNK2//DGKA//AGPAT4//PIP5K1C//MTM1//PIP5K1A//SYNJ1//IDI2//TIMP2//PRKAA2// |
| GO:0046165 | alcohol biosynthetic process | Biological process | 4 | 140 | 143 | 17653 | 3.52707292707293 | 0.0271664010299627 | 0.758840028400589 | 1.56596789254847 | 0.027972027972028 | DHCR24//PRKAA2//IDI2//PCK1// |
| GO:0070665 | positive regulation of leukocyte proliferation | Biological process | 4 | 140 | 143 | 17653 | 3.52707292707293 | 0.0271664010299627 | 0.758840028400589 | 1.56596789254847 | 0.027972027972028 | TFRC//CD274//CD209//HLA-DPB1// |
| GO:0002694 | regulation of leukocyte activation | Biological process | 9 | 525 | 143 | 17653 | 2.11624375624376 | 0.0272225417533757 | 0.758840028400589 | 1.56507132740858 | 0.0629370629370629 | TFRC//MAP3K8//CD274//HLA-DPB1//VNN1//CD209//ZC3H12A//THBS1//RIPK3// |
| GO:0031669 | cellular response to nutrient levels | Biological process | 5 | 208 | 143 | 17653 | 2.9674892415277 | 0.0272389133412017 | 0.758840028400589 | 1.56481022199175 | 0.034965034965035 | NCOA1//FAS//PRKAA2//ZC3H12A//PCK1// |
| GO:1901700 | response to oxygen-containing compound | Biological process | 20 | 1565 | 143 | 17653 | 1.57760450412208 | 0.0274881267708136 | 0.758840028400589 | 1.56085485494803 | 0.13986013986014 | GPX3//ATP6V0E1//THBS1//PRDX2//GJB2//FAS//LTA//PCK1//RARB//TRPC3//BAK1//NPPC//CD274//ZC3H12A//NOD1//GCLM//PRKAA2//SMO//KAT7//KIF1B// |
| GO:0002714 | positive regulation of B cell mediated immunity | Biological process | 2 | 32 | 143 | 17653 | 7.71547202797203 | 0.0275781652998877 | 0.758840028400589 | 1.5594346296264 | 0.013986013986014 | LTA//TFRC// |
| GO:0002891 | positive regulation of immunoglobulin mediated immune response | Biological process | 2 | 32 | 143 | 17653 | 7.71547202797203 | 0.0275781652998877 | 0.758840028400589 | 1.5594346296264 | 0.013986013986014 | LTA//TFRC// |
| GO:0042987 | amyloid precursor protein catabolic process | Biological process | 2 | 32 | 143 | 17653 | 7.71547202797203 | 0.0275781652998877 | 0.758840028400589 | 1.5594346296264 | 0.013986013986014 | FKBP1A//DHCR24// |
| GO:0097659 | nucleic acid-templated transcription | Biological process | 42 | 3924 | 143 | 17653 | 1.32130407818481 | 0.0276263427216668 | 0.758840028400589 | 1.55867660480224 | 0.293706293706294 | CUX2//NR6A1//MITF//DLL4//H2AFY2//PURB//RARB//SMO//NCOA1//EBF3//YBX2//ALX4//HIF3A//TAF11//FUBP3//CREB5//KAT7//FOXP4//ZNF488//ZNF597//ZNF320//KDM2A//ALX3//HOXA9//AFF3//PAX7//PRKAA2//RNF4//ZNF329//SLTM//ZC3H12A//NCOA4//ZNF251//LZTR1//WDHD1//QRICH1//CDK12//TMEM100//NOD1//RIPK3//PCK1//CUL2// |
| GO:0055076 | transition metal ion homeostasis | Biological process | 4 | 141 | 143 | 17653 | 3.50205822546248 | 0.0277893240138642 | 0.758840028400589 | 1.55612201747665 | 0.027972027972028 | TMEM199//TFRC//NCOA4//SLC30A2// |
| GO:0022607 | cellular component assembly | Biological process | 33 | 2934 | 143 | 17653 | 1.38846940380683 | 0.0280586596787062 | 0.758840028400589 | 1.55193307842685 | 0.230769230769231 | H2AFY2//CDH24//TNS1//FKBP1A//COX18//DZIP1//STX1A//SYNJ1//GJB2//PMP22//PIP5K1C//RIPK3//KCNC4//KCNS1//KCTD15//WDR1//PIP5K1A//CLTCL1//TAF11//NOD1//ZC3H12A//GPX3//ACPP//MCCC2//TMEM170A//THBS1//CUX2//FAS//MITF//TMEM199//RNF4//MTM1//SPAG17// |
| GO:0060415 | muscle tissue morphogenesis | Biological process | 3 | 81 | 143 | 17653 | 4.57213157213157 | 0.0280718806646459 | 0.758840028400589 | 1.55172849100225 | 0.020979020979021 | DLL4//FKBP1A//PAX7// |
| GO:0051259 | protein complex oligomerization | Biological process | 9 | 528 | 143 | 17653 | 2.10421964399237 | 0.0280964426461511 | 0.758840028400589 | 1.55134866361616 | 0.0629370629370629 | RIPK3//KCNC4//KCNS1//KCTD15//GPX3//ACPP//MCCC2//NOD1//ZC3H12A// |
| GO:1902532 | negative regulation of intracellular signal transduction | Biological process | 9 | 528 | 143 | 17653 | 2.10421964399237 | 0.0280964426461511 | 0.758840028400589 | 1.55134866361616 | 0.0629370629370629 | THBS1//MTM1//PRKAA2//MAPKBP1//ZC3H12A//TIMP2//MUL1//RASSF2//VNN1// |
| GO:0006650 | glycerophospholipid metabolic process | Biological process | 7 | 362 | 143 | 17653 | 2.38710736777035 | 0.0281633664244489 | 0.758840028400589 | 1.55031543432469 | 0.048951048951049 | ETNK2//DGKA//AGPAT4//PIP5K1C//MTM1//PIP5K1A//SYNJ1// |
| GO:0010628 | positive regulation of gene expression | Biological process | 23 | 1877 | 143 | 17653 | 1.51267645513783 | 0.0284823491561858 | 0.76263107254763 | 1.54542419398551 | 0.160839160839161 | NCOA1//LARP4B//THBS1//EBF3//MITF//RNF4//NCOA4//FUBP3//CREB5//KAT7//NR6A1//HOXA9//PAX7//CDK12//RARB//ALX4//HIF3A//SMO//ZC3H12A//PCK1//KIF1B//CUX2//DLL4// |
| GO:0050867 | positive regulation of cell activation | Biological process | 7 | 363 | 143 | 17653 | 2.38053131441561 | 0.0285295965332767 | 0.76263107254763 | 1.54470437010986 | 0.048951048951049 | TFRC//MAP3K8//CD274//HLA-DPB1//VNN1//CD209//THBS1// |
| GO:0030522 | intracellular receptor signaling pathway | Biological process | 6 | 285 | 143 | 17653 | 2.5988958410011 | 0.0286802653388345 | 0.763541869230205 | 1.54241683505938 | 0.041958041958042 | NCOA1//NR6A1//RNF4//NCOA4//RARB//NOD1// |
| GO:0055080 | cation homeostasis | Biological process | 11 | 707 | 143 | 17653 | 1.92068327711892 | 0.0288686826633631 | 0.763541869230205 | 1.53957303340661 | 0.0769230769230769 | TMEM199//TFRC//NCOA4//ATP6V0E1//TRPC5//BAK1//TRPC3//SLC41A1//FKBP1A//F2RL2//SLC30A2// |
| GO:0046849 | bone remodeling | Biological process | 3 | 82 | 143 | 17653 | 4.51637387003241 | 0.0289696655123449 | 0.763541869230205 | 1.5380565191898 | 0.020979020979021 | TFRC//MITF//RASSF2// |
| GO:0035264 | multicellular organism growth | Biological process | 4 | 143 | 143 | 17653 | 3.45307839014133 | 0.029060657190544 | 0.763541869230205 | 1.53669456859961 | 0.027972027972028 | NPPC//SMO//ETNK2//RARB// |
| GO:0071549 | cellular response to dexamethasone stimulus | Biological process | 2 | 33 | 143 | 17653 | 7.48166984530621 | 0.0292040563811456 | 0.763541869230205 | 1.53455682178457 | 0.013986013986014 | GJB2//PCK1// |
| GO:0070663 | regulation of leukocyte proliferation | Biological process | 5 | 212 | 143 | 17653 | 2.91149887848001 | 0.0292410681843299 | 0.763541869230205 | 1.53400676652653 | 0.034965034965035 | TFRC//CD274//CD209//HLA-DPB1//RIPK3// |
| GO:2001056 | positive regulation of cysteine-type endopeptidase activity | Biological process | 4 | 144 | 143 | 17653 | 3.42909867909868 | 0.029709101293992 | 0.770316659327093 | 1.52711048548895 | 0.027972027972028 | NOD1//FAS//MUL1//BAK1// |
| GO:2001234 | negative regulation of apoptotic signaling pathway | Biological process | 5 | 213 | 143 | 17653 | 2.89782986966086 | 0.0297557743782696 | 0.770316659327093 | 1.52642874296823 | 0.034965034965035 | FAS//VNN1//GCLM//THBS1//GFRAL// |
| GO:0034333 | adherens junction assembly | Biological process | 3 | 83 | 143 | 17653 | 4.46195972701997 | 0.0298825628928155 | 0.770316659327093 | 1.52458215777781 | 0.020979020979021 | PIP5K1A//THBS1//PIP5K1C// |
| GO:0006661 | phosphatidylinositol biosynthetic process | Biological process | 4 | 145 | 143 | 17653 | 3.4054497226911 | 0.0303660847003263 | 0.770316659327093 | 1.51761120092533 | 0.027972027972028 | SYNJ1//PIP5K1C//MTM1//PIP5K1A// |
| GO:0007269 | neurotransmitter secretion | Biological process | 4 | 145 | 143 | 17653 | 3.4054497226911 | 0.0303660847003263 | 0.770316659327093 | 1.51761120092533 | 0.027972027972028 | STX1A//PIP5K1C//SYNJ1//KCNC4// |
| GO:0099643 | signal release from synapse | Biological process | 4 | 145 | 143 | 17653 | 3.4054497226911 | 0.0303660847003263 | 0.770316659327093 | 1.51761120092533 | 0.027972027972028 | STX1A//PIP5K1C//SYNJ1//KCNC4// |
| GO:0055065 | metal ion homeostasis | Biological process | 10 | 624 | 143 | 17653 | 1.97832616101847 | 0.0306286592346688 | 0.770316659327093 | 1.51387201395835 | 0.0699300699300699 | TMEM199//TFRC//NCOA4//TRPC5//BAK1//TRPC3//SLC41A1//FKBP1A//F2RL2//SLC30A2// |
| GO:0051930 | regulation of sensory perception of pain | Biological process | 2 | 34 | 143 | 17653 | 7.26162073220897 | 0.0308673391509156 | 0.770316659327093 | 1.51050080627692 | 0.013986013986014 | ACPP//TMEM100// |
| GO:0051931 | regulation of sensory perception | Biological process | 2 | 34 | 143 | 17653 | 7.26162073220897 | 0.0308673391509156 | 0.770316659327093 | 1.51050080627692 | 0.013986013986014 | ACPP//TMEM100// |
| GO:0061384 | heart trabecula morphogenesis | Biological process | 2 | 34 | 143 | 17653 | 7.26162073220897 | 0.0308673391509156 | 0.770316659327093 | 1.51050080627692 | 0.013986013986014 | DLL4//FKBP1A// |
| GO:0070232 | regulation of T cell apoptotic process | Biological process | 2 | 34 | 143 | 17653 | 7.26162073220897 | 0.0308673391509156 | 0.770316659327093 | 1.51050080627692 | 0.013986013986014 | RIPK3//CD274// |
| GO:1904591 | positive regulation of protein import | Biological process | 2 | 34 | 143 | 17653 | 7.26162073220897 | 0.0308673391509156 | 0.770316659327093 | 1.51050080627692 | 0.013986013986014 | SMO//ZC3H12A// |
| GO:0031401 | positive regulation of protein modification process | Biological process | 16 | 1188 | 143 | 17653 | 1.66259329895694 | 0.0311253492047291 | 0.773899767174937 | 1.50688576738742 | 0.111888111888112 | MAP3K8//NOD1//THBS1//FAS//CDK2AP1//BAK1//TRAF4//MUL1//TRPC5//PAX7//FKBP1A//RASSF2//RIPK3//TIMP2//KAT7//ZC3H12A// |
| GO:0031326 | regulation of cellular biosynthetic process | Biological process | 48 | 4640 | 143 | 17653 | 1.27704364600916 | 0.0315744043269457 | 0.779042521555893 | 1.5006648339622 | 0.335664335664336 | CUX2//NR6A1//MITF//DLL4//H2AFY2//PURB//RARB//SMO//NCOA1//KAT7//ZNF597//ZNF320//KDM2A//AFF3//YBX2//PRKAA2//TAF11//ZNF329//LZTR1//ZNF251//WDHD1//FOXP4//ALX3//QRICH1//HIF3A//SLTM//CREB5//LARP4B//THBS1//EBF3//RNF4//NCOA4//FUBP3//ZNF488//TMEM100//NPPC//NOD1//ZC3H12A//NECAB3//TIMP2//HOXA9//PAX7//CDK12//ALX4//RIPK3//PCK1//CUL2//NMD3// |
| GO:0009187 | cyclic nucleotide metabolic process | Biological process | 4 | 147 | 143 | 17653 | 3.35911707340279 | 0.0317057264108272 | 0.779042521555893 | 1.49886229222943 | 0.027972027972028 | NPPC//PDE1B//THBS1//TIMP2// |
| GO:0080090 | regulation of primary metabolic process | Biological process | 63 | 6394 | 143 | 17653 | 1.21632715110976 | 0.0318940829756428 | 0.779042521555893 | 1.49628988024635 | 0.440559440559441 | CUX2//NR6A1//MITF//DLL4//H2AFY2//PURB//RARB//SMO//MAP3K8//NOD1//THBS1//NCOA1//FKBP1A//ZC3H12A//FAS//CDK2AP1//KAT7//ZNF597//ZNF320//KDM2A//AFF3//YBX2//PRKAA2//TAF11//ZNF329//LZTR1//ZNF251//WDHD1//FOXP4//ALX3//QRICH1//HIF3A//SLTM//CREB5//MUL1//BAK1//TRAF4//USP51//TRPC5//TIMP2//LARP4B//EBF3//RNF4//NCOA4//FUBP3//ZNF488//TMEM100//NPPC//PAX7//RASSF2//RIPK3//MTM1//PDE1B//NECAB3//DHCR24//CDK12//TFRC//HOXA9//ALX4//NOVA2//PCK1//CUL2//NMD3// |
| GO:0098771 | inorganic ion homeostasis | Biological process | 11 | 720 | 143 | 17653 | 1.88600427350427 | 0.0323469376075447 | 0.779042521555893 | 1.49016682915601 | 0.0769230769230769 | TMEM199//TFRC//NCOA4//ATP6V0E1//TRPC5//BAK1//TRPC3//SLC41A1//FKBP1A//F2RL2//SLC30A2// |
| GO:0003230 | cardiac atrium development | Biological process | 2 | 35 | 143 | 17653 | 7.05414585414585 | 0.0325673102779898 | 0.779042521555893 | 1.48721810817333 | 0.013986013986014 | DLL4//SMO// |
| GO:0048713 | regulation of oligodendrocyte differentiation | Biological process | 2 | 35 | 143 | 17653 | 7.05414585414585 | 0.0325673102779898 | 0.779042521555893 | 1.48721810817333 | 0.013986013986014 | ZNF488//WDR1// |
| GO:0051204 | protein insertion into mitochondrial membrane | Biological process | 2 | 35 | 143 | 17653 | 7.05414585414585 | 0.0325673102779898 | 0.779042521555893 | 1.48721810817333 | 0.013986013986014 | COX18//PPP1R13B// |
| GO:1901655 | cellular response to ketone | Biological process | 3 | 86 | 143 | 17653 | 4.30630996910067 | 0.0327116610667121 | 0.779042521555893 | 1.48529740225866 | 0.020979020979021 | PRKAA2//GJB2//PCK1// |
| GO:0010557 | positive regulation of macromolecule biosynthetic process | Biological process | 21 | 1698 | 143 | 17653 | 1.52673651436902 | 0.0329046081245968 | 0.779042521555893 | 1.48274327703656 | 0.146853146853147 | NCOA1//LARP4B//THBS1//EBF3//MITF//RNF4//NCOA4//FUBP3//CREB5//KAT7//NR6A1//HOXA9//PAX7//CDK12//RARB//ALX4//HIF3A//SMO//ZC3H12A//PCK1//NMD3// |
| GO:0097191 | extrinsic apoptotic signaling pathway | Biological process | 5 | 219 | 143 | 17653 | 2.81843727049207 | 0.0329642532726855 | 0.779042521555893 | 1.48195675772478 | 0.034965034965035 | BAK1//FAS//THBS1//GCLM//GFRAL// |
| GO:0060341 | regulation of cellular localization | Biological process | 12 | 815 | 143 | 17653 | 1.81763267407439 | 0.033158299194937 | 0.779042521555893 | 1.47940775403242 | 0.0839160839160839 | STX1A//BAK1//TRPC3//SMO//ZC3H12A//KCNC4//FKBP1A//KAT7//PPP1R13B//NBPF3//SHISA6//NMD3// |
| GO:0042221 | response to chemical | Biological process | 48 | 4655 | 143 | 17653 | 1.27292857518421 | 0.0333021193250276 | 0.779042521555893 | 1.47752812739827 | 0.335664335664336 | NCOA1//PDE1B//THBS1//GPX3//BAK1//FKBP1A//COX4I1//GCLM//LTA//ATP6V0E1//TIMP2//DHCR24//RARB//SLC30A2//RNF4//GJB2//STX1A//PRDX2//TMEM100//NR6A1//NCOA4//PIP5K1C//FAS//PCK1//TRPC3//CD274//ZC3H12A//AFF3//PRKAA2//TFRC//DLL4//TMEM199//NPPC//PIP5K1A//HLA-DPB1//MUL1//HIF3A//CUL2//NOD1//MAP3K8//SLC41A1//CUX2//PTPN7//SMO//KAT7//VNN1//KIF1B//LAPTM5// |
| GO:0032092 | positive regulation of protein binding | Biological process | 3 | 87 | 143 | 17653 | 4.25681215336388 | 0.0336847259006342 | 0.779042521555893 | 1.47256698223817 | 0.020979020979021 | TRAF4//FKBP1A//NMD3// |
| GO:0045981 | positive regulation of nucleotide metabolic process | Biological process | 3 | 87 | 143 | 17653 | 4.25681215336388 | 0.0336847259006342 | 0.779042521555893 | 1.47256698223817 | 0.020979020979021 | NPPC//TIMP2//PRKAA2// |
| GO:0048644 | muscle organ morphogenesis | Biological process | 3 | 87 | 143 | 17653 | 4.25681215336388 | 0.0336847259006342 | 0.779042521555893 | 1.47256698223817 | 0.020979020979021 | DLL4//FKBP1A//PAX7// |
| GO:0055072 | iron ion homeostasis | Biological process | 3 | 87 | 143 | 17653 | 4.25681215336388 | 0.0336847259006342 | 0.779042521555893 | 1.47256698223817 | 0.020979020979021 | TMEM199//TFRC//NCOA4// |
| GO:0090559 | regulation of membrane permeability | Biological process | 3 | 87 | 143 | 17653 | 4.25681215336388 | 0.0336847259006342 | 0.779042521555893 | 1.47256698223817 | 0.020979020979021 | BAK1//PPP1R13B//MUL1// |
| GO:1900544 | positive regulation of purine nucleotide metabolic process | Biological process | 3 | 87 | 143 | 17653 | 4.25681215336388 | 0.0336847259006342 | 0.779042521555893 | 1.47256698223817 | 0.020979020979021 | NPPC//TIMP2//PRKAA2// |
| GO:0043066 | negative regulation of apoptotic process | Biological process | 13 | 911 | 143 | 17653 | 1.76160063865882 | 0.0337459775603005 | 0.779042521555893 | 1.47177798661001 | 0.0909090909090909 | ZC3H12A//DHCR24//THBS1//GCLM//GFRAL//MITF//FAS//VNN1//PAX7//PRKAA2//RARB//SMO//PRDX2// |
| GO:0001890 | placenta development | Biological process | 4 | 150 | 143 | 17653 | 3.29193473193473 | 0.0337795644136663 | 0.779042521555893 | 1.47134595494147 | 0.027972027972028 | GJB2//ST14//NCOA1//ETNK2// |
| GO:0019882 | antigen processing and presentation | Biological process | 5 | 221 | 143 | 17653 | 2.7929310508496 | 0.0340799053040736 | 0.779042521555893 | 1.46750162065344 | 0.034965034965035 | THBS1//NOD1//HLA-DPB1//CLEC4M//CD209// |
| GO:0019432 | triglyceride biosynthetic process | Biological process | 2 | 36 | 143 | 17653 | 6.85819735819736 | 0.0343032752312389 | 0.779042521555893 | 1.46466441211622 | 0.013986013986014 | PCK1//MOGAT3// |
| GO:0033572 | transferrin transport | Biological process | 2 | 36 | 143 | 17653 | 6.85819735819736 | 0.0343032752312389 | 0.779042521555893 | 1.46466441211622 | 0.013986013986014 | TFRC//ATP6V0E1// |
| GO:0070266 | necroptotic process | Biological process | 2 | 36 | 143 | 17653 | 6.85819735819736 | 0.0343032752312389 | 0.779042521555893 | 1.46466441211622 | 0.013986013986014 | RIPK3//FAS// |
| GO:0051960 | regulation of nervous system development | Biological process | 12 | 820 | 143 | 17653 | 1.80654954801296 | 0.0345072140868199 | 0.779042521555893 | 1.46209010172156 | 0.0839160839160839 | SMO//PMP22//RARB//TIMP2//NCOA1//TRPC5//WDR1//ZNF488//DLL4//CUX2//LTA//MUL1// |
| GO:0007568 | aging | Biological process | 6 | 298 | 143 | 17653 | 2.48552119021918 | 0.0345506111082244 | 0.779042521555893 | 1.46154426670997 | 0.041958041958042 | TPRA1//GJB2//GCLM//PCK1//BAK1//TIMP2// |
| GO:0008544 | epidermis development | Biological process | 8 | 462 | 143 | 17653 | 2.13761995580177 | 0.0345644610388211 | 0.779042521555893 | 1.46137021065167 | 0.0559440559440559 | ALX4//ST14//PIP5K1A//SMO//KRT6C//KRTAP4-4//H2AFY2//TCHH// |
| GO:0071453 | cellular response to oxygen levels | Biological process | 5 | 222 | 143 | 17653 | 2.78035028035028 | 0.0346464476174446 | 0.779042521555893 | 1.46034128801411 | 0.034965034965035 | TMEM199//HIF3A//CUL2//FAS//PCK1// |
| GO:0016079 | synaptic vesicle exocytosis | Biological process | 3 | 88 | 143 | 17653 | 4.20843928798474 | 0.0346727486305373 | 0.779042521555893 | 1.46001172893031 | 0.020979020979021 | STX1A//SYNJ1//PIP5K1C// |
| GO:0060255 | regulation of macromolecule metabolic process | Biological process | 63 | 6425 | 143 | 17653 | 1.21045849092542 | 0.0352118453235873 | 0.786088658814091 | 1.45331121455346 | 0.440559440559441 | CUX2//NR6A1//MITF//DLL4//H2AFY2//PURB//RARB//SMO//MAP3K8//NOD1//THBS1//ZC3H12A//NCOA1//FKBP1A//FAS//CDK2AP1//KAT7//ZNF597//ZNF320//KDM2A//AFF3//YBX2//PRKAA2//TAF11//ZNF329//LZTR1//ZNF251//WDHD1//FOXP4//ALX3//QRICH1//HIF3A//SLTM//CREB5//MUL1//BAK1//TRAF4//USP51//KIF1B//FUBP3//TRPC5//TIMP2//LARP4B//EBF3//RNF4//NCOA4//ZNF488//TMEM100//PAX7//RASSF2//RIPK3//MTM1//NECAB3//DHCR24//CDK12//TFRC//HOXA9//ALX4//NPPC//NOVA2//PCK1//CUL2//NMD3// |
| GO:0043122 | regulation of I-kappaB kinase/NF-kappaB signaling | Biological process | 5 | 223 | 143 | 17653 | 2.76788234187337 | 0.0352188176283705 | 0.786088658814091 | 1.45322522832598 | 0.034965034965035 | NOD1//FKBP1A//MUL1//MAPKBP1//ZC3H12A// |
| GO:0007044 | cell-substrate junction assembly | Biological process | 3 | 89 | 143 | 17653 | 4.16115345328829 | 0.0356756905631817 | 0.786338896987132 | 1.44762761162953 | 0.020979020979021 | PIP5K1A//THBS1//TNS1// |
| GO:0043410 | positive regulation of MAPK cascade | Biological process | 9 | 552 | 143 | 17653 | 2.01273183338401 | 0.0358113606075009 | 0.786338896987132 | 1.44597917820328 | 0.0629370629370629 | MAP3K8//NOD1//THBS1//MUL1//TRAF4//RASSF2//ZC3H12A//FAS//TIMP2// |
| GO:0008285 | negative regulation of cell proliferation | Biological process | 11 | 732 | 143 | 17653 | 1.85508617065994 | 0.0358147504307984 | 0.786338896987132 | 1.44593807080453 | 0.0769230769230769 | THBS1//CD274//LTA//DLL4//DHCR24//NPPC//PMP22//BAK1//RARB//TIMP2//CUL2// |
| GO:0042149 | cellular response to glucose starvation | Biological process | 2 | 37 | 143 | 17653 | 6.67284067284067 | 0.0360745481848427 | 0.786338896987132 | 1.44279909951537 | 0.013986013986014 | PRKAA2//ZC3H12A// |
| GO:0046839 | phospholipid dephosphorylation | Biological process | 2 | 37 | 143 | 17653 | 6.67284067284067 | 0.0360745481848427 | 0.786338896987132 | 1.44279909951537 | 0.013986013986014 | MTM1//SYNJ1// |
| GO:0009966 | regulation of signal transduction | Biological process | 35 | 3215 | 143 | 17653 | 1.34390803597647 | 0.0364138638646872 | 0.786338896987132 | 1.43873323575761 | 0.244755244755245 | MAP3K8//NOD1//THBS1//NCOA1//TRAF4//MUL1//RGS7//LTA//NPPC//PIP5K1C//PIP5K1A//MTM1//PRKAA2//TIMP2//FAS//FKBP1A//F2RL2//MAPKBP1//ZC3H12A//CDK12//DLL4//SMO//RASSF2//TMEM100//ACPP//SHISA6//BAK1//PPP1R13B//TAF11//VNN1//CUX2//STX1A//GCLM//GFRAL//RIPK3// |
| GO:0030216 | keratinocyte differentiation | Biological process | 6 | 302 | 143 | 17653 | 2.4526003797527 | 0.0365022384591649 | 0.786338896987132 | 1.43768050210093 | 0.041958041958042 | KRT6C//KRTAP4-4//H2AFY2//ST14//TCHH//PIP5K1A// |
| GO:1903037 | regulation of leukocyte cell-cell adhesion | Biological process | 6 | 302 | 143 | 17653 | 2.4526003797527 | 0.0365022384591649 | 0.786338896987132 | 1.43768050210093 | 0.041958041958042 | MAP3K8//CD274//HLA-DPB1//VNN1//CD209//TFRC// |
| GO:0006139 | nucleobase-containing compound metabolic process | Biological process | 59 | 5967 | 143 | 17653 | 1.22061431111205 | 0.0370487512409059 | 0.786338896987132 | 1.43122642569608 | 0.412587412587413 | KAT7//CUX2//NR6A1//MITF//DLL4//H2AFY2//PURB//RARB//SMO//ZC3H12A//DBR1//NOVA2//NCOA1//EXOG//EBF3//YBX2//ALX4//HIF3A//TAF11//FUBP3//CREB5//H6PD//COX4I1//ACPP//NPPC//PDE1B//WDHD1//CDK2AP1//USP51//KDM2A//FOXP4//ZNF488//ZNF597//ZNF320//ALX3//HOXA9//AFF3//PAX7//PRKAA2//RNF4//ZNF329//SLTM//NCOA4//ZNF251//LZTR1//QRICH1//CDK12//BAK1//MAPKBP1//MCCC2//TMEM100//THBS1//NOD1//TIMP2//TFRC//RIPK3//PCK1//CUL2//NMD3// |
| GO:0043069 | negative regulation of programmed cell death | Biological process | 13 | 924 | 143 | 17653 | 1.73681621408894 | 0.0371700086812293 | 0.786338896987132 | 1.42980733747276 | 0.0909090909090909 | DHCR24//FAS//PAX7//PRKAA2//RARB//SMO//PRDX2//THBS1//ZC3H12A//GCLM//GFRAL//MITF//VNN1// |
| GO:2001236 | regulation of extrinsic apoptotic signaling pathway | Biological process | 4 | 155 | 143 | 17653 | 3.1857432889691 | 0.0374082895675759 | 0.786338896987132 | 1.42703214874768 | 0.027972027972028 | FAS//THBS1//GCLM//GFRAL// |
| GO:0006644 | phospholipid metabolic process | Biological process | 8 | 470 | 143 | 17653 | 2.10123493527749 | 0.0376484392349079 | 0.786338896987132 | 1.42425302333359 | 0.0559440559440559 | ETNK2//PIP5K1A//DGKA//AGPAT4//PIP5K1C//MTM1//SYNJ1//IDI2// |
| GO:0034220 | ion transmembrane transport | Biological process | 15 | 1119 | 143 | 17653 | 1.65479292825137 | 0.0376643053147052 | 0.786338896987132 | 1.42407003834085 | 0.104895104895105 | PRKAA2//BAK1//THBS1//ATP6V0E1//KCNC4//CACNA2D4//SLC41A1//FKBP1A//WDR1//TRPC3//TRPC5//SLC30A2//KCNS1//COX4I1//SHISA6// |
| GO:0006767 | water-soluble vitamin metabolic process | Biological process | 3 | 91 | 143 | 17653 | 4.06969953123799 | 0.0377261650752452 | 0.786338896987132 | 1.42335733928102 | 0.020979020979021 | MCCC2//ACPP//VNN1// |
| GO:0030316 | osteoclast differentiation | Biological process | 3 | 91 | 143 | 17653 | 4.06969953123799 | 0.0377261650752452 | 0.786338896987132 | 1.42335733928102 | 0.020979020979021 | MITF//RASSF2//TFRC// |
| GO:0046460 | neutral lipid biosynthetic process | Biological process | 2 | 38 | 143 | 17653 | 6.49723960250276 | 0.0378804519244613 | 0.786338896987132 | 1.42158484838236 | 0.013986013986014 | MOGAT3//PCK1// |
| GO:0046463 | acylglycerol biosynthetic process | Biological process | 2 | 38 | 143 | 17653 | 6.49723960250276 | 0.0378804519244613 | 0.786338896987132 | 1.42158484838236 | 0.013986013986014 | MOGAT3//PCK1// |
| GO:0050691 | regulation of defense response to virus by host | Biological process | 2 | 38 | 143 | 17653 | 6.49723960250276 | 0.0378804519244613 | 0.786338896987132 | 1.42158484838236 | 0.013986013986014 | ZC3H12A//MUL1// |
| GO:0098815 | modulation of excitatory postsynaptic potential | Biological process | 2 | 38 | 143 | 17653 | 6.49723960250276 | 0.0378804519244613 | 0.786338896987132 | 1.42158484838236 | 0.013986013986014 | CUX2//STX1A// |
| GO:1901030 | positive regulation of mitochondrial outer membrane permeabilization involved in apoptotic signaling pathway | Biological process | 2 | 38 | 143 | 17653 | 6.49723960250276 | 0.0378804519244613 | 0.786338896987132 | 1.42158484838236 | 0.013986013986014 | PPP1R13B//BAK1// |
| GO:0034612 | response to tumor necrosis factor | Biological process | 6 | 305 | 143 | 17653 | 2.4284764415912 | 0.0380116370897181 | 0.786338896987132 | 1.42008342574758 | 0.041958041958042 | FAS//LTA//PCK1//THBS1//ZC3H12A//AFF3// |
| GO:0099531 | presynaptic process involved in chemical synaptic transmission | Biological process | 4 | 156 | 143 | 17653 | 3.16532185762955 | 0.0381599496778537 | 0.786338896987132 | 1.41839220667876 | 0.027972027972028 | STX1A//PIP5K1C//SYNJ1//KCNC4// |
| GO:0002520 | immune system development | Biological process | 13 | 928 | 143 | 17653 | 1.72932993730408 | 0.0382720440700768 | 0.786338896987132 | 1.41711834193938 | 0.0909090909090909 | BAK1//RIPK3//LTA//DLL4//WDR1//PDE1B//MITF//TFRC//VNN1//PURB//HOXA9//THBS1//RASSF2// |
| GO:0010556 | regulation of macromolecule biosynthetic process | Biological process | 46 | 4467 | 143 | 17653 | 1.2712306721709 | 0.0384658254987507 | 0.786338896987132 | 1.41492494291672 | 0.321678321678322 | CUX2//NR6A1//MITF//DLL4//H2AFY2//PURB//RARB//SMO//NCOA1//KAT7//ZNF597//ZNF320//KDM2A//AFF3//YBX2//PRKAA2//TAF11//ZNF329//LZTR1//ZNF251//WDHD1//FOXP4//ALX3//QRICH1//HIF3A//SLTM//CREB5//LARP4B//THBS1//EBF3//RNF4//NCOA4//FUBP3//ZNF488//TMEM100//NOD1//ZC3H12A//NECAB3//HOXA9//PAX7//CDK12//ALX4//RIPK3//PCK1//CUL2//NMD3// |
| GO:0043009 | chordate embryonic development | Biological process | 9 | 560 | 143 | 17653 | 1.98397852147852 | 0.0386791736242962 | 0.786338896987132 | 1.41252281313717 | 0.0629370629370629 | ETNK2//TMEM100//SMO//ST14//PAX7//ALX3//ALX4//HOXA9//NCOA1// |
| GO:0051254 | positive regulation of RNA metabolic process | Biological process | 19 | 1523 | 143 | 17653 | 1.54005482370551 | 0.0387079283020688 | 0.786338896987132 | 1.41220007205631 | 0.132867132867133 | NCOA1//EBF3//MITF//RNF4//NCOA4//FUBP3//CREB5//KAT7//NR6A1//HOXA9//PAX7//CDK12//RARB//ALX4//HIF3A//SMO//ZC3H12A//PCK1//NMD3// |
| GO:2000379 | positive regulation of reactive oxygen species metabolic process | Biological process | 3 | 92 | 143 | 17653 | 4.02546366676801 | 0.0387736082525101 | 0.786338896987132 | 1.4114637818858 | 0.020979020979021 | RIPK3//THBS1//ZC3H12A// |
| GO:0071496 | cellular response to external stimulus | Biological process | 6 | 307 | 143 | 17653 | 2.41265574816063 | 0.039039805057723 | 0.786338896987132 | 1.40849235961617 | 0.041958041958042 | NCOA1//FAS//BAK1//PRKAA2//ZC3H12A//PCK1// |
| GO:0010562 | positive regulation of phosphorus metabolic process | Biological process | 15 | 1126 | 143 | 17653 | 1.64450558322672 | 0.0394395615949702 | 0.786338896987132 | 1.40406792126489 | 0.104895104895105 | MAP3K8//NOD1//THBS1//FAS//CDK2AP1//BAK1//TRAF4//MUL1//TRPC5//RIPK3//NPPC//RASSF2//TIMP2//PRKAA2//ZC3H12A// |
| GO:0045937 | positive regulation of phosphate metabolic process | Biological process | 15 | 1126 | 143 | 17653 | 1.64450558322672 | 0.0394395615949702 | 0.786338896987132 | 1.40406792126489 | 0.104895104895105 | MAP3K8//NOD1//THBS1//FAS//CDK2AP1//BAK1//TRAF4//MUL1//TRPC5//RIPK3//NPPC//RASSF2//TIMP2//PRKAA2//ZC3H12A// |
| GO:1901362 | organic cyclic compound biosynthetic process | Biological process | 48 | 4704 | 143 | 17653 | 1.25966890252605 | 0.0394755024345395 | 0.786338896987132 | 1.40367233369055 | 0.335664335664336 | CUX2//NR6A1//MITF//DLL4//H2AFY2//PURB//RARB//SMO//NCOA1//EBF3//YBX2//ALX4//HIF3A//TAF11//FUBP3//CREB5//NPPC//PDE1B//KAT7//FOXP4//ZNF488//ZNF597//ZNF320//KDM2A//ALX3//HOXA9//AFF3//PAX7//PRKAA2//RNF4//ZNF329//SLTM//ZC3H12A//NCOA4//ZNF251//LZTR1//WDHD1//QRICH1//CDK12//DHCR24//IDI2//TMEM100//NOD1//TIMP2//RIPK3//PCK1//CUL2//NMD3// |
| GO:0001505 | regulation of neurotransmitter levels | Biological process | 6 | 308 | 143 | 17653 | 2.404822450277 | 0.0395604882342011 | 0.786338896987132 | 1.40273835721361 | 0.041958041958042 | STX1A//PIP5K1C//SYNJ1//PDE1B//ZC3H12A//KCNC4// |
| GO:0003205 | cardiac chamber development | Biological process | 4 | 158 | 143 | 17653 | 3.1252544923431 | 0.0396892230399 | 0.786338896987132 | 1.40132740280074 | 0.027972027972028 | RARB//DLL4//FKBP1A//SMO// |
| GO:0015682 | ferric iron transport | Biological process | 2 | 39 | 143 | 17653 | 6.3306437152591 | 0.0397203177543385 | 0.786338896987132 | 1.4009872858966 | 0.013986013986014 | TFRC//ATP6V0E1// |
| GO:0021532 | neural tube patterning | Biological process | 2 | 39 | 143 | 17653 | 6.3306437152591 | 0.0397203177543385 | 0.786338896987132 | 1.4009872858966 | 0.013986013986014 | PAX7//SMO// |
| GO:0046006 | regulation of activated T cell proliferation | Biological process | 2 | 39 | 143 | 17653 | 6.3306437152591 | 0.0397203177543385 | 0.786338896987132 | 1.4009872858966 | 0.013986013986014 | CD274//RIPK3// |
| GO:0071377 | cellular response to glucagon stimulus | Biological process | 2 | 39 | 143 | 17653 | 6.3306437152591 | 0.0397203177543385 | 0.786338896987132 | 1.4009872858966 | 0.013986013986014 | GJB2//PCK1// |
| GO:0072512 | trivalent inorganic cation transport | Biological process | 2 | 39 | 143 | 17653 | 6.3306437152591 | 0.0397203177543385 | 0.786338896987132 | 1.4009872858966 | 0.013986013986014 | TFRC//ATP6V0E1// |
| GO:0050865 | regulation of cell activation | Biological process | 9 | 563 | 143 | 17653 | 1.97340669987206 | 0.0397940169703702 | 0.786338896987132 | 1.40018221918455 | 0.0629370629370629 | TFRC//MAP3K8//CD274//HLA-DPB1//VNN1//CD209//ZC3H12A//THBS1//RIPK3// |
| GO:0009889 | regulation of biosynthetic process | Biological process | 48 | 4707 | 143 | 17653 | 1.25886605427714 | 0.0398808578539977 | 0.786338896987132 | 1.39923550840325 | 0.335664335664336 | CUX2//NR6A1//MITF//DLL4//H2AFY2//PURB//RARB//SMO//NCOA1//KAT7//ZNF597//ZNF320//KDM2A//AFF3//YBX2//PRKAA2//TAF11//ZNF329//LZTR1//ZNF251//WDHD1//FOXP4//ALX3//QRICH1//HIF3A//SLTM//CREB5//LARP4B//THBS1//EBF3//RNF4//NCOA4//FUBP3//ZNF488//TMEM100//NPPC//NOD1//ZC3H12A//NECAB3//TIMP2//HOXA9//PAX7//CDK12//ALX4//RIPK3//PCK1//CUL2//NMD3// |
| GO:0042327 | positive regulation of phosphorylation | Biological process | 14 | 1032 | 143 | 17653 | 1.6746760990947 | 0.0404024002092065 | 0.791584755438414 | 1.39359283373469 | 0.0979020979020979 | MAP3K8//NOD1//THBS1//FAS//CDK2AP1//BAK1//TRAF4//MUL1//TRPC5//RASSF2//RIPK3//TIMP2//PRKAA2//ZC3H12A// |
| GO:0051094 | positive regulation of developmental process | Biological process | 17 | 1329 | 143 | 17653 | 1.57908833078133 | 0.0405694920422802 | 0.791584755438414 | 1.39180042992485 | 0.118881118881119 | SMO//VNN1//ZC3H12A//TMEM100//H2AFY2//RARB//TIMP2//NCOA1//NPPC//THBS1//TRPC5//TFRC//MTM1//ZNF488//CUX2//LTA//MUL1// |
| GO:0045785 | positive regulation of cell adhesion | Biological process | 7 | 392 | 143 | 17653 | 2.20442057942058 | 0.0405952613125133 | 0.791584755438414 | 1.39152465868726 | 0.048951048951049 | EDIL3//MAP3K8//CD274//HLA-DPB1//VNN1//CD209//TFRC// |
| GO:0010506 | regulation of autophagy | Biological process | 6 | 310 | 143 | 17653 | 2.38930746672682 | 0.0406150983494203 | 0.791584755438414 | 1.39131249078471 | 0.041958041958042 | PRKAA2//ZC3H12A//ATP6V0E1//MTM1//NOD1//MUL1// |
| GO:0030097 | hemopoiesis | Biological process | 12 | 842 | 143 | 17653 | 1.75934754082022 | 0.0409120098708391 | 0.792814781141269 | 1.38814918453551 | 0.0839160839160839 | BAK1//DLL4//WDR1//PDE1B//MITF//TFRC//RIPK3//VNN1//PURB//HOXA9//THBS1//RASSF2// |
| GO:0009953 | dorsal/ventral pattern formation | Biological process | 3 | 94 | 143 | 17653 | 3.93981550364529 | 0.0409126657723352 | 0.792814781141269 | 1.38814222198056 | 0.020979020979021 | PAX7//SMO//DLL4// |
| GO:0034654 | nucleobase-containing compound biosynthetic process | Biological process | 46 | 4488 | 143 | 17653 | 1.26528240030914 | 0.0413677249026781 | 0.797961377679817 | 1.38333836330517 | 0.321678321678322 | CUX2//NR6A1//MITF//DLL4//H2AFY2//PURB//RARB//SMO//NCOA1//EBF3//YBX2//ALX4//HIF3A//TAF11//FUBP3//CREB5//NPPC//PDE1B//KAT7//FOXP4//ZNF488//ZNF597//ZNF320//KDM2A//ALX3//HOXA9//AFF3//PAX7//PRKAA2//RNF4//ZNF329//SLTM//ZC3H12A//NCOA4//ZNF251//LZTR1//WDHD1//QRICH1//CDK12//TMEM100//NOD1//TIMP2//RIPK3//PCK1//CUL2//NMD3// |
| GO:0043030 | regulation of macrophage activation | Biological process | 2 | 40 | 143 | 17653 | 6.17237762237762 | 0.0415934854053283 | 0.797961377679817 | 1.38097468557692 | 0.013986013986014 | ZC3H12A//THBS1// |
| GO:2000112 | regulation of cellular macromolecule biosynthetic process | Biological process | 45 | 4376 | 143 | 17653 | 1.26945609235372 | 0.0417203352830181 | 0.797961377679817 | 1.37965221004945 | 0.314685314685315 | CUX2//NR6A1//MITF//DLL4//H2AFY2//PURB//RARB//SMO//NCOA1//KAT7//ZNF597//ZNF320//KDM2A//AFF3//YBX2//PRKAA2//TAF11//ZNF329//LZTR1//ZNF251//WDHD1//FOXP4//ALX3//QRICH1//HIF3A//SLTM//CREB5//LARP4B//THBS1//EBF3//RNF4//NCOA4//FUBP3//ZNF488//TMEM100//NOD1//ZC3H12A//NECAB3//HOXA9//PAX7//CDK12//ALX4//RIPK3//PCK1//CUL2// |
| GO:0009755 | hormone-mediated signaling pathway | Biological process | 5 | 234 | 143 | 17653 | 2.63776821469129 | 0.0419026817121746 | 0.797961377679817 | 1.37775818191364 | 0.034965034965035 | NCOA1//NR6A1//RNF4//NCOA4//RARB// |
| GO:0016925 | protein sumoylation | Biological process | 3 | 95 | 143 | 17653 | 3.89834376150166 | 0.0420041772074545 | 0.797961377679817 | 1.37670751798451 | 0.020979020979021 | RNF4//MUL1//MITF// |
| GO:1990823 | response to leukemia inhibitory factor | Biological process | 3 | 95 | 143 | 17653 | 3.89834376150166 | 0.0420041772074545 | 0.797961377679817 | 1.37670751798451 | 0.020979020979021 | GCLM//TFRC//LAPTM5// |
| GO:1990830 | cellular response to leukemia inhibitory factor | Biological process | 3 | 95 | 143 | 17653 | 3.89834376150166 | 0.0420041772074545 | 0.797961377679817 | 1.37670751798451 | 0.020979020979021 | GCLM//TFRC//LAPTM5// |
| GO:0002521 | leukocyte differentiation | Biological process | 8 | 483 | 143 | 17653 | 2.04467995772344 | 0.0430468669650698 | 0.810429162008717 | 1.36605845185949 | 0.0559440559440559 | BAK1//DLL4//PDE1B//MITF//TFRC//RIPK3//VNN1//RASSF2// |
| GO:0051251 | positive regulation of lymphocyte activation | Biological process | 6 | 315 | 143 | 17653 | 2.35138195138195 | 0.0433292769883138 | 0.810429162008717 | 1.36321855773704 | 0.041958041958042 | TFRC//MAP3K8//CD274//HLA-DPB1//VNN1//CD209// |
| GO:0006654 | phosphatidic acid biosynthetic process | Biological process | 2 | 41 | 143 | 17653 | 6.02183182670988 | 0.0434993029438362 | 0.810429162008717 | 1.36151770235734 | 0.013986013986014 | DGKA//AGPAT4// |
| GO:0045026 | plasma membrane fusion | Biological process | 2 | 41 | 143 | 17653 | 6.02183182670988 | 0.0434993029438362 | 0.810429162008717 | 1.36151770235734 | 0.013986013986014 | LYZL6//STX1A// |
| GO:0048488 | synaptic vesicle endocytosis | Biological process | 2 | 41 | 143 | 17653 | 6.02183182670988 | 0.0434993029438362 | 0.810429162008717 | 1.36151770235734 | 0.013986013986014 | SYNJ1//PIP5K1C// |
| GO:0097300 | programmed necrotic cell death | Biological process | 2 | 41 | 143 | 17653 | 6.02183182670988 | 0.0434993029438362 | 0.810429162008717 | 1.36151770235734 | 0.013986013986014 | RIPK3//FAS// |
| GO:1903587 | regulation of blood vessel endothelial cell proliferation involved in sprouting angiogenesis | Biological process | 2 | 41 | 143 | 17653 | 6.02183182670988 | 0.0434993029438362 | 0.810429162008717 | 1.36151770235734 | 0.013986013986014 | DLL4//THBS1// |
| GO:0031668 | cellular response to extracellular stimulus | Biological process | 5 | 237 | 143 | 17653 | 2.60437874361925 | 0.0438499312486113 | 0.814717266577907 | 1.35803108321863 | 0.034965034965035 | NCOA1//PRKAA2//FAS//ZC3H12A//PCK1// |
| GO:0010522 | regulation of calcium ion transport into cytosol | Biological process | 3 | 97 | 143 | 17653 | 3.81796553961502 | 0.0442308922159952 | 0.819543901525412 | 1.35427430004764 | 0.020979020979021 | BAK1//TRPC3//FKBP1A// |
| GO:0016241 | regulation of macroautophagy | Biological process | 4 | 164 | 143 | 17653 | 3.01091591335494 | 0.0444847822820367 | 0.821996127249766 | 1.35178853061986 | 0.027972027972028 | PRKAA2//MTM1//NOD1//ATP6V0E1// |
| GO:0032502 | developmental process | Biological process | 61 | 6267 | 143 | 17653 | 1.20157981479188 | 0.0450974864642255 | 0.825001592852774 | 1.345847663114 | 0.426573426573427 | CDH24//ALX4//RASSF2//MCAM//DLL4//TMEM100//HIF3A//ZC3H12A//SMO//RARB//ETNK2//ST14//THBS1//BAK1//FKBP1A//NPPC//EDIL3//DZIP1//CUX2//EBF3//HOXA9//FAS//QRICH1//KCTD15//FOXP4//PAX7//LZTR1//CLTCL1//ALX3//DHCR24//GFRAL//TRPC5//TIMP2//H2AFY2//PMP22//RIPK3//LTA//TPRA1//GJB2//GCLM//PCK1//WDR1//NCOA4//MUL1//ZNF488//TACC1//PURB//PIP5K1A//PDE1B//MITF//TFRC//TRAF4//KRT6C//KRTAP4-4//VNN1//AFF3//NCOA1//YBX2//MTM1//TCHH//CDK12// |
| GO:0030804 | positive regulation of cyclic nucleotide biosynthetic process | Biological process | 2 | 42 | 143 | 17653 | 5.87845487845488 | 0.0454371266816656 | 0.825001592852774 | 1.34258914001166 | 0.013986013986014 | NPPC//TIMP2// |
| GO:0043124 | negative regulation of I-kappaB kinase/NF-kappaB signaling | Biological process | 2 | 42 | 143 | 17653 | 5.87845487845488 | 0.0454371266816656 | 0.825001592852774 | 1.34258914001166 | 0.013986013986014 | MAPKBP1//ZC3H12A// |
| GO:0046473 | phosphatidic acid metabolic process | Biological process | 2 | 42 | 143 | 17653 | 5.87845487845488 | 0.0454371266816656 | 0.825001592852774 | 1.34258914001166 | 0.013986013986014 | DGKA//AGPAT4// |
| GO:0090051 | negative regulation of cell migration involved in sprouting angiogenesis | Biological process | 2 | 42 | 143 | 17653 | 5.87845487845488 | 0.0454371266816656 | 0.825001592852774 | 1.34258914001166 | 0.013986013986014 | DLL4//THBS1// |
| GO:0045893 | positive regulation of transcription, DNA-templated | Biological process | 18 | 1450 | 143 | 17653 | 1.532452375211 | 0.0455013446893516 | 0.825001592852774 | 1.34197576856188 | 0.125874125874126 | NCOA1//KAT7//EBF3//NR6A1//HOXA9//MITF//PAX7//CDK12//RARB//RNF4//ALX4//HIF3A//SMO//ZC3H12A//FUBP3//PCK1//NCOA4//CREB5// |
| GO:1903508 | positive regulation of nucleic acid-templated transcription | Biological process | 18 | 1450 | 143 | 17653 | 1.532452375211 | 0.0455013446893516 | 0.825001592852774 | 1.34197576856188 | 0.125874125874126 | NCOA1//EBF3//MITF//RNF4//NCOA4//FUBP3//CREB5//KAT7//NR6A1//HOXA9//PAX7//CDK12//RARB//ALX4//HIF3A//SMO//ZC3H12A//PCK1// |
| GO:0009792 | embryo development ending in birth or egg hatching | Biological process | 9 | 578 | 143 | 17653 | 1.92219372323179 | 0.0456974515178228 | 0.826341883997421 | 1.34010801924324 | 0.0629370629370629 | ETNK2//TMEM100//SMO//ST14//PAX7//ALX3//ALX4//HOXA9//NCOA1// |
| GO:0051260 | protein homooligomerization | Biological process | 6 | 320 | 143 | 17653 | 2.31464160839161 | 0.0461551707258864 | 0.830926845307003 | 1.33577963825827 | 0.041958041958042 | GPX3//ACPP//RIPK3//KCNC4//KCNS1//KCTD15// |
| GO:0008283 | cell proliferation | Biological process | 24 | 2078 | 143 | 17653 | 1.42576576455306 | 0.0461967313079156 | 0.830926845307003 | 1.3353887522611 | 0.167832167832168 | THBS1//SMO//NPPC//RARB//TRPC5//DHCR24//PMP22//DLL4//BAK1//TIMP2//CUL2//TFRC//FKBP1A//CD274//CD209//HLA-DPB1//FAS//MITF//RIPK3//LTA//PAX7//NR6A1//PURB//TACC1// |
| GO:0002832 | negative regulation of response to biotic stimulus | Biological process | 2 | 43 | 143 | 17653 | 5.74174662546756 | 0.047406321086763 | 0.841493305799943 | 1.32416374629909 | 0.013986013986014 | MUL1//MAPKBP1// |
| GO:0045104 | intermediate filament cytoskeleton organization | Biological process | 2 | 43 | 143 | 17653 | 5.74174662546756 | 0.047406321086763 | 0.841493305799943 | 1.32416374629909 | 0.013986013986014 | MTM1//KRT6C// |
| GO:0050798 | activated T cell proliferation | Biological process | 2 | 43 | 143 | 17653 | 5.74174662546756 | 0.047406321086763 | 0.841493305799943 | 1.32416374629909 | 0.013986013986014 | RIPK3//CD274// |
| GO:0071548 | response to dexamethasone | Biological process | 2 | 43 | 143 | 17653 | 5.74174662546756 | 0.047406321086763 | 0.841493305799943 | 1.32416374629909 | 0.013986013986014 | GJB2//PCK1// |
| GO:1901224 | positive regulation of NIK/NF-kappaB signaling | Biological process | 2 | 43 | 143 | 17653 | 5.74174662546756 | 0.047406321086763 | 0.841493305799943 | 1.32416374629909 | 0.013986013986014 | TRAF4//NOD1// |
| GO:0009790 | embryo development | Biological process | 13 | 959 | 143 | 17653 | 1.67342876102 | 0.0476165058205629 | 0.841521841260679 | 1.32224247702281 | 0.0909090909090909 | ETNK2//TMEM100//SMO//ST14//DLL4//PAX7//ALX3//HOXA9//ALX4//AFF3//RARB//TPRA1//NCOA1// |
| GO:0021510 | spinal cord development | Biological process | 3 | 100 | 143 | 17653 | 3.70342657342657 | 0.0476793144973378 | 0.841521841260679 | 1.32166999723098 | 0.020979020979021 | PAX7//SMO//DLL4// |
| GO:0010822 | positive regulation of mitochondrion organization | Biological process | 4 | 168 | 143 | 17653 | 2.93922743922744 | 0.047854834976016 | 0.841521841260679 | 1.32007417706531 | 0.027972027972028 | MUL1//BAK1//PPP1R13B//NBPF3// |
| GO:0003007 | heart morphogenesis | Biological process | 5 | 243 | 143 | 17653 | 2.54007309562865 | 0.0479056496947156 | 0.841521841260679 | 1.31961326557185 | 0.034965034965035 | SMO//RARB//TMEM100//DLL4//FKBP1A// |
| GO:0006066 | alcohol metabolic process | Biological process | 6 | 324 | 143 | 17653 | 2.28606578606579 | 0.0484969032211134 | 0.847008157023164 | 1.31428599246827 | 0.041958041958042 | MOGAT3//DHCR24//PRKAA2//IDI2//SYNJ1//PCK1// |
| GO:0042127 | regulation of cell proliferation | Biological process | 20 | 1668 | 143 | 17653 | 1.48018648018648 | 0.0485627203636301 | 0.847008157023164 | 1.31369699314734 | 0.13986013986014 | THBS1//SMO//RARB//TRPC5//DHCR24//NPPC//PMP22//DLL4//BAK1//TIMP2//CUL2//TFRC//CD274//CD209//HLA-DPB1//RIPK3//LTA//PAX7//FAS//MITF// |
| GO:0045637 | regulation of myeloid cell differentiation | Biological process | 5 | 244 | 143 | 17653 | 2.52966295999083 | 0.0486025611367728 | 0.847008157023164 | 1.31334084476526 | 0.034965034965035 | HOXA9//THBS1//MITF//RASSF2//PURB// |
| GO:0046328 | regulation of JNK cascade | Biological process | 4 | 169 | 143 | 17653 | 2.92183556088882 | 0.0487189373180557 | 0.847008157023164 | 1.31230219332136 | 0.027972027972028 | MUL1//NOD1//TRAF4//RASSF2// |
| GO:0048598 | embryonic morphogenesis | Biological process | 9 | 586 | 143 | 17653 | 1.8959521706962 | 0.0490745231420548 | 0.848057176531154 | 1.30914391175405 | 0.0629370629370629 | ST14//SMO//DLL4//ALX3//HOXA9//ALX4//AFF3//RARB//NCOA1// |
| GO:0001818 | negative regulation of cytokine production | Biological process | 5 | 245 | 143 | 17653 | 2.51933780505209 | 0.049305471325432 | 0.848057176531154 | 1.30710488529478 | 0.034965034965035 | CD274//THBS1//ZC3H12A//MUL1//MAPKBP1// |
| GO:0031018 | endocrine pancreas development | Biological process | 2 | 44 | 143 | 17653 | 5.61125238397966 | 0.0494062586948506 | 0.848057176531154 | 1.30621803195758 | 0.013986013986014 | SMO//BAK1// |
| GO:0045103 | intermediate filament-based process | Biological process | 2 | 44 | 143 | 17653 | 5.61125238397966 | 0.0494062586948506 | 0.848057176531154 | 1.30621803195758 | 0.013986013986014 | KRT6C//MTM1// |
| GO:0048599 | oocyte development | Biological process | 2 | 44 | 143 | 17653 | 5.61125238397966 | 0.0494062586948506 | 0.848057176531154 | 1.30621803195758 | 0.013986013986014 | NPPC//YBX2// |
| GO:0071347 | cellular response to interleukin-1 | Biological process | 4 | 170 | 143 | 17653 | 2.90464829288359 | 0.0495916620935244 | 0.849084584148115 | 1.30459133583171 | 0.027972027972028 | NOD1//MAP3K8//PCK1//ZC3H12A// |
